# Assessing the Association Between Respiratory Symptoms and Nicotine and Cannabis Use Through Traditional and E-Product Devices in the U.S.

**DOI:** 10.1016/j.focus.2024.100291

**Published:** 2024-10-22

**Authors:** Phil Veliz, John Jardine, Luisa Kcomt, Carol Boyd, Sean Esteban McCabe, Rebecca Evans-Polce

**Affiliations:** 1 Center for the Study of Alcohol, Smoking and Health, School of Nursing, University of Michigan, Ann Arbor, Michigan; 2 School of Social Work, Wayne State University, Detroit, Michigan

**Keywords:** Nicotine use, e-products, cigarettes, cannabis, vaping, Population Assessment of Tobacco and Health (PATH) Study

## Abstract

**Introduction:**

The aim of this study was to assess the association between past 30-day cigarette smoking, nicotine use with an e-product device (e.g., vape), cannabis smoking, cannabis use with an e-product device, and other forms of cannabis use and past-year respiratory symptoms in a nationally representative sample of people aged ≥12 years in the U.S. during 2021.

**Methods:**

Data from Wave 6 (2021) of the Population Assessment of Tobacco and Health Study, a national probability sample of adolescents (aged 12–17 years; *n*=5,652) and adults (aged ≥18 years; *n*=30,516), were used. Logistic regression was used to assess the association of past 30-day cigarette smoking, nicotine use with an e-product, cannabis smoking, cannabis use with an e-product, and other forms of cannabis use with past-year respiratory symptoms (a past-year respiratory symptom index was constructed and ranged from 0 to 9; an index of 2+ was flagged as having functionally important respiratory impairment).

**Results:**

The odds of a past-year respiratory index score of 2+ was greater among respondents who indicated either past 30-day cigarette or cannabis smoking than among respondents who did not use any nicotine or cannabis during the past 30 days. Past 30-day nicotine and cannabis use with an e-product device was not associated with a respiratory symptom index of 2+. The combination of past 30-day cigarette smoking and cannabis smoking had one of the strongest associations with experiencing a respiratory index score of 2+ among dual users of nicotine and cannabis.

**Conclusions:**

Past 30-day cigarette smoking and cannabis smoking were associated with higher odds of functionally important respiratory impairment. In addition, this study provides robust evidence regarding the potential respiratory harms of single or dual nicotine and cannabis smoking as it relates to respiratory health.

## HIGHLIGHTS

• Cigarette and cannabis smoking is associated with respiratory impairment.
• Only vaping nicotine or cannabis was not associated with respiratory impairment.
• Cessation services should be tailored for nicotine and cannabis use that involves combustibles.

## INTRODUCTION

Cannabis use has increased substantially within the U.S. population over the past several years among both adolescents and adults.^1^, ^2^, ^3^ In particular, the use of cannabis in e-products (i.e., vaping) has increased 5-fold among adolescents in the U.S. population since 2013.^4^ The Centers for Disease Control and Prevention found that E-cigarette or vaping use–associated lung injury^5^ was predominately associated with vaping cannabis products (vitamin E acetate with tetrahydrocannabinol in vaping liquids^6^^,^^7^). According to the Centers for Disease Control and Prevention, 84% of the cases were associated with cannabis-containing products, whereas 16% were among those using only nicotine-containing products.^5^

Several studies conducted in the U.S. have found that cannabis use in e-products is associated with various types of respiratory problems in both adolescents and adults.^8^, ^9^, ^10^ However, most of these studies assessing respiratory problems have not examined how single e-product cannabis use compares with multiproduct use (e.g., dual use) with other nicotine and cannabis products within the U.S. population.^8^, ^9^, ^10^, ^11^, ^12^, ^13^, ^14^, ^15^, ^16^, ^17^, ^18^ At this time, relatively little is known about the population-level respiratory health consequences of single or dual nicotine and cannabis use as it relates to both combustible and e-product use.

It should be recognized that both nicotine and cannabis products have rapidly evolved and have become more accessible in the past decade.^19^ Indeed, newer-generation e-product devices deliver a higher concentration of nicotine and cannabis than older delivery systems.^20^, ^21^, ^22^ Although the prevalence of cigarette smoking has decreased over the past half century, the use of e-product devices for nicotine or cannabis has gained popularity, particularly among adolescents and young adults.^6^^,^^23^^,^^24^ Concomitant with changes in potency, trends in nicotine and cannabis use have changed, making it increasingly important to understand the health risks associated with single and dual use of nicotine and cannabis and whether it is being consumed using e-products or combustible products. Accordingly, to fill this knowledge gap, the authors investigated the association between past 30-day cigarette smoking, nicotine use with an e-product device (e.g., vape), cannabis smoking, cannabis use with an e-product device, and other forms of cannabis use and past-year respiratory symptoms among U.S. individuals aged ≥12 years during 2021.

## METHODS

### Study Sample

The authors used data from a national probability sample of adolescents and adults (aged ≥12 years) from the Population Assessment of Tobacco and Health (PATH) Study: Wave 6 (March 1, 2021–November 30, 2021).^25^ The analytic sample at Wave 6 included 30,516 adults who were aged ≥18 years and 5,652 youth who were between the ages of 12 and 17 years (Table 1 provides demographic and operational definitions). The weighted response rate for the youth and adult Wave 1 cohort at Wave 6 were 56.6% and 57.5%, respectively, and the weighted response rate for the youth and adult Wave 4 cohort was 63.6% and 73.5%, respectively.^25^ The University of Michigan Human Subjects Review Board determined study exemption.

**Table 1:** Estimated Distributions of Key Study Measures for Participants Aged ≥12 Years in the PATH, Wave 6 (N=36,168)

Demographics	*n*	% (95% CI)
Sex^a^		
Male	17,519	46.69 (46.22, 47.15)
Female	18,590	53.31 (52.85, 53.78)
Race^b^		
White	24,333	76.48 (75.93, 77.02)
Black	5,799	12.73 (12.40, 13.05)
Other	4,581	10.80 (10.39, 11.22)
Age, years^c^		
12–17	5,652	2.86 (2.79, 2.92)
18–34	17,677	25.64 (25.15, 26.13)
35–54	6,640	31.26 (30.57, 31.96)
≥55	6,199	40.25 (39.56, 40.94)
Household income^d^		
≤$9,999	3,667	7.15 (6.69, 7.64)
$10,000–$24,999	5,282	13.62 (13.02, 14.24)
$25,000–$49,999	7,579	20.48 (19.70, 21.28)
$50,000–$99,999	8,919	28.23 (27.26, 29.22)
≥$100,000	8,522	28.03 (27.05, 29.03)
Nonresponse follow-up: >$50,000	567	1.39 (1.16, 1.66)
Nonresponse follow-up: <$50,000	502	1.10 (0.95, 1.29)
Lifetime substance use^e^		
Smoked cigarettes	19,384	63.13 (61.64, 64.60)
Used e-product (nicotine)	16,334	29.11 (28.30, 29.93)
Used other form(s) of tobacco	18,220	49.55 (48.19, 50.91)
Used cannabis in any way	18,280	45.55 (44.26, 46.85)
Past 30-day substance use^f^		
Smoked cigarettes	6,954	14.55 (14.05, 15.07)
Used e-product (nicotine)	4,338	6.46 (6.20, 6.73)
Used cannabis in any way	7,547	14.88 (14.23, 15.55)
Smoked cannabis	6,085	11.14 (10.64, 11.66)
Used cannabis in an e-product	2,517	4.32 (4.05, 4.60)
Used cannabis in some other way	1,623	3.98 (3.63, 4.36)
Lifetime self-reported respiratory symptoms		
Wheezing or whistling in chest	14,719	38.47 (37.52, 39.44)
Past-year self-reported respiratory symptoms		
Wheezing or whistling in chest	4,541	11.82 (11.25, 12.41)
Sleep disturbed due to wheezing	1,733	4.68 (4.35, 5.03)
Speech limited due to wheezing	777	1.87 (1.68, 2.08)
Sounded wheezy during or after exercise	3,759	8.70 (8.19, 9.25)
Dry cough at night not associated with cold/chest infection	5,609	15.94 (15.25, 16.66)
Respiratory symptom index (≥2)	6,735	17.55 (16.89, 18.24)

*Notes: n*=unweighted sample size; percentages and 95% CIs incorporate cross-sectional replicate weights (Wave 4 cohort).

a Sex of participant was a derived variable, that is, PATH Study constructed the variable from the interview; sex was coded as male or female.

b Race of participant was a derived variable from the interview and was coded as White alone, Black alone, or other.

c Age of respondent was a derived variable from the interview.

d Household income was a derived variable from the interview and was coded as <$10,000, $10,000–$24,999, $25,000–$49,999, $50,000–$99,999, or ≥$100,000. Participants who did not know or refused to report their household income were probed further to respond either >$50,000 or <$50,000.

e Lifetime cigarette, e-product (nicotine), and other tobacco use were coded from derived variables provided by the PATH at Wave 6. Other tobacco use included the following products: traditional cigar, cigarillo, filtered cigar, pipe, hookah, snus, smokeless tobacco, dissolvable tobacco, IQOS, bidi, and kretek. Lifetime cannabis use was coded from all lifetime and past 12-month Waves 1–6 questions asking *have you smoked part or all of a traditional cigar, cigarillo, or filtered cigar with marijuana in it*? or *have you used marijuana, hash, THC, grass, pot, or weed?*

f Past 30-day cigarette and e-product (nicotine) use were coded from derived variables provided by the PATH at Wave 6. Past 30-day cannabis use was coded from the question asking *have you used marijuana in the past 30 days?* (this measure also used the past 12-month and lifetime questions). Participants who used cannabis in the past 30 days were asked which of the 4 ways they used it: smoked dried herb or flower in a joint, pipe, hookah, or bong; smoked dried herb or flower in a blunt cigar, cigarillo, or filtered cigar; vaped marijuana liquids or oils in an E-cigarette, vape pen, or electronic nicotine product; or used marijuana some other way. The first 2 options were combined into 1 measure: smoked cannabis. IQOS, I quit ordinary smoking; PATH, Population Assessment of Tobacco and Health; THC, tetrahydrocannabinol.

### Measures

The dependent measures for the analysis included the following self-reported symptoms during the past year at Wave 6: (1) wheezing or whistling in the chest, (2) sleep disturbed owing to wheezing, (3) speech limited due to wheezing, (4) sounded wheezy during or after exercise, and (5) dry cough at night not associated with a cold or chest infection. Each symptom was treated as a binary variable (yes/no) and was examined separately. In addition, the authors calculated a past-year respiratory symptom index on the basis of 7 questions provided in the PATH that have been validated in prior research to assess respiratory symptom severity^17^^,^^26^: (1) *Have you ever had wheezing or whistling in the chest at any time in the past?* [*Yes*/*No*]; (2) *Have you had wheezing or whistling in the chest in the past 12 months?* [*Yes*/*No*]; (3) *How many attacks of wheezing have you had in the past 12 months?* [*None* or *1–3, 4–12, More than 12*]; (4) *In the past 12 months, how often, on average has your sleep been disturbed due to wheezing?* [*Never, Less than one night per week, One or more nights per week*]; (5) *In the past 12 months, has wheezing ever been severe enough to limit your speech to only one or two words between breaths?* [*Yes*/*No*]; (6) *In the past 12 months, has your chest sounded wheezy during or after exercise?* [*Yes*/*No*]; and (7) *In the past 12 months, have you had a dry cough at night?* [*Yes*/*No*]. The respiratory symptom index ranged from 0 to 9. A respiratory index score of 2 or higher was defined as an indicator of having functionally important respiratory symptoms on the basis of prior guidelines.^17^ Table 2, Table 3 (footnotes) provide more details on how each symptom was measured.

**Table 2:** Past-Year Self-Reported Respiratory Symptoms as a Function of Past 30-Day Substance Use Among U.S. Participants Aged ≥12 Years

	Wheezing or whistling in the chest^a^		Sleep disturbed owing to wheezing^b^		Speech limited owing to wheezing^c^	
Measure	%	AOR (95% CI)	%	AOR (95% CI)	%	AOR (95% CI)
Past 30-day substance use (mutually exclusive categories)		*n*=29,835		*n*=29,824		*n*=29,831
No use	8.28	ref	31.06	ref	1.34	ref
Cigarette smoking only	27.80	3.07 (2.60, 3.62)	12.09	2.44 (1.91, 3.12)	3.71	1.10 (0.77, 1.57)
Cannabis smoking only	13.41	1.68 (1.23, 2.30)	5.21	1.54 (0.95, 2.48)	1.83	1.11 (0.60, 2.03)
Nicotine use with e-product only	7.95	0.69 (0.50, 0.96)	3.10	0.68 (0.39, 1.20)	1.26	0.56 (0.24, 1.29)
Cigarette smoking and cannabis smoking	31.39	3.79 (3.00, 4.79)	13.72	2.86 (2.06, 3.97)	5.12	1.57 (1.00, 2.48)
Cigarette smoking and nicotine use with e-product	21.50	1.94 (1.38, 2.73)	11.29	2.08 (1.29, 3.35)	3.92	1.06 (0.46, 2.41)
Cannabis smoking and cannabis use with e-product	11.67	1.17 (0.73, 1.86)	3.86	0.96 (0.51, 1.81)	2.49	1.41 (0.73, 2.72)
Other cannabis use only	16.10	1.65 (1.08, 2.52)	7.25	2.01 (1.15, 3.52)	3.54	2.32 (0.97, 5.52)
Nicotine use with e-product and cannabis smoking	11.54	1.15 (0.76, 1.75)	2.60	0.64 (0.25, 1.61)	0.75	0.33 (0.08, 1.32)
Cigarette smoking, nicotine use with e-product, and cannabis smoking	27.23	2.63 (1.77, 3.91)	12.08	2.00 (1.16, 3.45)	5.44	1.54 (0.73, 3.27)
Nicotine use with e-product, cannabis smoking, and cannabis use with e-product	11.02	0.97 (0.57, 1.63)	2.72	0.48 (0.24, 1.00)	1.76	0.83 (0.29, 2.38)
Cannabis use with e-product only	12.75	1.65 (0.76, 3.59)	6.16	2.42 (0.72, 8.18)	1.85	1.45 (0.30, 6.93)
Cigarette smoking, nicotine use with e-product, cannabis smoking, and cannabis use with e-product	31.53	4.31 (2.72, 6.82)	16.25	4.19 (2.10, 8.34)	5.16	2.01 (0.89, 4.58)
Cigarette smoking, cannabis smoking, and cannabis use with e-product	31.05	3.14 (1.87, 5.26)	10.96	1.97 (1.07, 3.62)	4.78	1.37 (0.56, 3.36)
Cannabis smoking and other cannabis use	19.55	2.21 (1.28, 3.82)	9.15	2.65 (1.21, 5.82)	2.34	1.50 (0.49, 4.60)
Cannabis smoking, cannabis use with e-product, and other cannabis use	20.11	2.34 (1.09, 5.04)	5.08	1.34 (0.61, 2.98)	4.06	2.22 (0.90, 5.46)
Nicotine use with e-product and cannabis use with e-product	17.22	2.06 (1.01, 4.20)	1.63	0.42 (0.09, 2.03)	2.62	1.44 (0.22, 9.35)
Cigarette smoking and other cannabis use	41.97	4.36 (2.29, 8.27)	20.47	3.55 (1.68, 7.53)	7.98	1.87 (0.78, 4.48)
Cigarette smoking, cannabis smoking, and other cannabis use	40.24	4.85 (2.23, 10.53)	14.29	2.07 (0.78, 5.50)	9.55	2.20 (0.41, 11.89)
Nicotine use with e-product, cannabis smoking, cannabis use with e-product, and other cannabis use	25.25	3.03 (1.51, 6.10)	9.49	1.91 (0.79, 4.58)	9.24	3.64 (0.91, 14.55)
Cannabis use with e-product and other cannabis use	13.17	1.84 (0.72, 4.68)	6.19	2.51 (0.85, 7.37)	1.44	1.09 (0.08, 14.41)
Cigarette smoking and cannabis use with e-product	38.78	9.11 (2.68, 30.98)	7.77	2.14 (0.59, 7.85)	3.60	2.68 (0.55, 13.02)
Cigarette smoking, cannabis smoking, cannabis use with e-product, and other cannabis use	50.21	8.44 (3.77, 18.90)	15.96	3.03 (1.07, 8.59)	8.12	2.77 (0.55, 14.04)
Cigarette smoking, nicotine use with e-product, cannabis smoking, cannabis use with e-product, and other cannabis use	49.20	8.61 (2.93, 25.31)	29.58	8.25 (3.07, 22.17)	3.64	0.52 (0.08, 3.40)
Cigarette smoking, nicotine use with e-product, and cannabis use with e-product	18.69	1.64 (0.47, 5.75)	10.51	1.36 (0.14, 13.41)	1.72	0.37 (0.02, 7.02)
Nicotine use with e-product and other cannabis use	9.89	1.09 (0.35, 3.34)	4.55	1.36 (0.17, 10.69)	3.27	2.05 (0.16, 26.92)
Nicotine use with e-product, cannabis smoking, and other cannabis use	10.76	1.27 (0.46, 3.54)	2.73	0.78 (0.09, 6.94)	0.61	0.29 (0.02, 4.25)
Cigarette smoking, nicotine use with e-product, and other cannabis use	30.72	4.00 (1.60, 9.97)	16.23	3.78 (1.00, 14.31)	7.72	3.02 (0.55, 16.61)
Cigarette smoking, nicotine use with e-product, cannabis smoking, and other cannabis use	28.43	2.31 (0.60, 8.92)	9.85	1.07 (0.25, 4.59)	5.17	0.84 (0.10, 6.83)
Nicotine use with e-product, cannabis use with e-product, and other cannabis use	10.93	0.93 (0.10, 8.37)	0.00	0.00 (0.00, 3.70)	0.00	0.00 (0.00, 1.15)
Cigarette smoking, nicotine use with e-product, cannabis use with e-product, and other cannabis use	16.88	1.29 (0.03, 60.43)	0.00	0.00 (0.00, 2.54)	0.00	0.00 (0.00, 0.98)
Cigarette smoking, cannabis use with e-product, and other cannabis use	32.78	2.69 (0.43, 16.94)	19.74	2.40 (0.18, 32.27)	0.00	0.00 (0.00, 0.38)

*Notes:* Unweighted samples sizes are provided. Prevalence, AORs, and 95% CIs are weighted to be representative of the U.S. population. All models control for sex, race, age, and household income; lifetime uses of cigarettes, electronic nicotine products, other tobacco products, and marijuana; lifetime diagnoses of high blood pressure, high cholesterol, diabetes, bronchitis, and asthma (adults and youth); lifetime diagnoses of congestive heart failure, stroke, heart attack, other heart conditions, COPD, emphysema, and other respiratory conditions; and use of beta blockers (adults only).

a This item was measured with the following question: *Have you had wheezing or whistling in the chest in the past 12 months?* Response options were *Yes* and *No*.

b This item was measured with the following question: *In the past 12 months, how often, on average has your sleep been disturbed due to wheezing?* Response options were *Never woken with wheezing, Less than one night per week*, and *One or more nights per week*. This item was recoded as a binary variable.

c This item was measured with the following question: *In the past 12 months, has wheezing ever been severe enough to limit your speech to only one or two words between breaths?* Response options were *Yes* and *No*. COPD, chronic obstructive pulmonary disease.

**Table 3:** Past-Year Self-Reported Respiratory Symptoms as a Function of Past 30-Day Substance Use Among U.S. Participants Aged ≥12 Years

	Sounded wheezy during or after exercise^a^		Dry cough at night not associated with cold/chest infection^b^		Respiratory symptom index (≥2)	
Measure	%	AOR (95% CI)	%	AOR (95% CI)	%	AOR (95% CI)
Past 30-day substance use (mutually exclusive categories)		*n*=29,828		*n*=29,832		*n*=29,766
No use	6.30	ref	13.52	ref	12.95	ref
Cigarette smoking only	16.38	1.92 (1.58, 2.34)	27.27	1.91 (1.66, 2.20)	36.96	3.01 (2.63, 3.46)
Cannabis smoking only	10.10	1.32 (0.95, 1.82)	15.46	1.07 (0.83, 1.38)	19.42	1.52 (1.17, 1.97)
Nicotine use with e-product only	9.67	1.02 (0.76, 1.36)	11.84	0.84 (0.64, 1.09)	15.25	0.92 (0.71, 1.20)
Cigarette smoking and cannabis smoking	20.47	2.41 (1.84, 3.15)	28.98	2.07 (1.68, 2.55)	42.38	3.85 (3.11, 4.77)
Cigarette smoking and nicotine use with e-product	16.57	1.47 (1.04, 2.07)	22.34	1.51 (1.17, 1.96)	30.49	1.89 (1.40, 2.56)
Cannabis smoking and cannabis use with e-product	14.50	1.68 (1.04, 2.72)	17.18	1.23 (0.83, 1.81)	22.29	1.53 (1.00, 2.34)
Other cannabis use only	12.13	1.48 (0.86, 2.56)	22.97	1.65 (1.08, 2.53)	25.89	1.88 (1.27, 2.78)
Nicotine use with e-product and cannabis smoking	11.09	1.19 (0.76, 1.88)	17.04	1.37 (0.97, 1.93)	19.72	1.38 (0.94, 2.01)
Cigarette smoking, nicotine use with e-product, and cannabis smoking	23.45	2.33 (1.59, 3.42)	29.75	2.15 (1.55, 3.00)	38.07	2.66 (1.84, 3.86)
Nicotine use with e-product, cannabis smoking, and cannabis use with e-product	9.58	0.80 (0.48, 1.34)	18.18	1.38 (0.97, 1.95)	20.15	1.25 (0.83, 1.88)
Cannabis use with e-product only	12.84	1.79 (0.85, 3.76)	16.02	1.27 (0.75, 2.15)	18.12	1.42 (0.78, 2.56)
Cigarette smoking, nicotine use with e-product, cannabis smoking, and cannabis use with e-product	23.60	2.58 (1.58, 4.23)	32.65	2.84 (1.89, 4.25)	42.17	4.24 (2.75, 6.54)
Cigarette smoking, cannabis smoking, and cannabis use with e-product	18.66	1.88 (1.10, 3.21)	27.66	1.92 (1.29, 2.88)	41.75	3.20 (1.93, 5.30)
Cannabis smoking and other cannabis use	14.46	1.84 (1.09, 3.09)	18.05	1.36 (0.90, 2.04)	24.72	1.81 (1.17, 2.79)
Cannabis smoking, cannabis use with e-product, and other cannabis use	20.98	2.62 (1.24, 5.54)	16.16	1.17 (0.70, 1.93)	30.98	2.62 (1.35, 5.09)
Nicotine use with e-product and cannabis use with e-product	15.84	1.77 (0.83, 3.76)	22.57	1.91 (1.01, 3.61)	25.01	1.84 (0.97, 3.50)
Cigarette smoking and other cannabis use	29.74	3.10 (1.81, 5.31)	28.15	1.50 (0.85, 2.65)	51.01	3.81 (2.10, 6.93)
Cigarette smoking, cannabis smoking, and other cannabis use	19.28	1.44 (0.81, 2.59)	24.38	1.51 (0.68, 3.35)	43.68	3.25 (1.51, 7.01)
Nicotine use with e-product, cannabis smoking, cannabis use with e-product, and other cannabis use	19.66	1.87 (0.76, 4.65)	27.56	2.29 (1.20, 4.35)	31.43	2.51 (1.25, 5.06)
Cannabis use with e-product and other cannabis use	11.13	1.70 (0.78, 3.69)	23.54	2.24 (0.83, 6.06)	25.00	2.76 (1.00, 7.59)
Cigarette smoking and cannabis use with e-product	34.52	7.66 (2.06, 28.45)	47.98	6.19 (2.37, 16.22)	47.01	7.67 (2.64, 22.31)
Cigarette smoking, cannabis smoking, cannabis use with e-product, and other cannabis use	45.63	8.23 (3.84, 17.62)	30.93	2.09 (0.89, 4.92)	63.06	8.72 (4.33, 17.55)
Cigarette smoking, nicotine use with e-product, cannabis smoking, cannabis use with e-product, and other cannabis use	33.53	3.68 (1.24, 10.96)	31.09	1.93 (0.87, 4.30)	58.84	7.38 (3.01, 18.09)
Cigarette smoking, nicotine use with e-product, and cannabis use with e-product	19.06	1.99 (0.63, 6.31)	22.03	0.92 (0.31, 2.70)	33.15	2.57 (0.95, 6.99)
Nicotine use with e-product and other cannabis use	8.67	1.00 (0.32, 3.11)	20.99	1.80 (0.43, 7.53)	13.31	0.90 (0.34, 2.41)
Nicotine use with e-product, cannabis smoking, and other cannabis use	14.05	2.02 (0.55, 7.45)	16.21	1.08 (0.35, 3.34)	27.21	2.32 (0.70, 7.67)
Cigarette smoking, nicotine use with e-product, and other cannabis use	19.68	2.11 (0.78, 5.77)	27.59	2.14 (0.86, 5.30)	39.07	3.31 (1.48, 7.41)
Cigarette smoking, nicotine use with e-product, cannabis smoking, and other cannabis use	18.29	1.18 (0.45, 3.08)	25.08	1.37 (0.38, 4.93)	39.59	2.32 (0.79, 6.76)
Nicotine use with e-product, cannabis use with e-product, and other cannabis use	10.93	0.83 (0.09, 7.59)	16.63	1.32 (0.08, 21.57)	10.93	0.51 (0.05, 5.05)
Cigarette smoking, nicotine use with e-product, cannabis use with e-product, and other cannabis use	11.14	0.97 (0.03, 32.69)	0.00	0.00 (0.00, 3.77)	16.88	0.80 (0.02, 34.07)
Cigarette smoking, cannabis use with e-product, and other cannabis use	19.74	1.11 (0.12, 10.17)	7.96	0.31 (0.01, 7.08)	32.78	1.44 (0.22, 9.57)

*Notes:* Unweighted samples sizes are provided. Prevalence, AORs, and 95% CIs are weighted to be representative of the U.S. population. All models control for sex, race, age, and household income; lifetime uses of cigarettes, electronic nicotine products, other tobacco products, and marijuana; lifetime diagnoses of high blood pressure, high cholesterol, diabetes, bronchitis, and asthma (adults and youth); lifetime diagnoses of congestive heart failure, stroke, heart attack, other heart conditions, COPD, emphysema, and other respiratory conditions; and use of beta blockers (adults only).

a This item was measured with the following question: *In the past 12 months, has your chest sounded wheezy during or after exercise?* Response options were *Yes* and *No*.

b This item was measured with the following question: *A dry cough is a cough without phlegm or mucus. In the past 12 months, have you had a dry cough at night?* Response options were *Yes* and *No*. COPD, chronic obstructive pulmonary disease.

The key independent variables were past 30-day cigarette smoking, past 30-day nicotine use with an e-product, past 30-day cannabis smoking (without an e-product), past 30-day cannabis use with an e-product, and past 30-day other form(s) of cannabis use. Past 30-day cigarette and e-product nicotine use were based on derived variables provided by the PATH at Wave 6. For determining methods of cannabis use, participants who answered *Yes* to the question *Have you used marijuana within the past 30 days?* were asked which of the 4 ways they used it: (1) *Smoke dried herb or flower in a joint, pipe, hookah, or bong*; (2) *Smoke dried herb or flower in a blunt cigar, cigarillo, or filtered cigar*; (3) *Vape marijuana liquids or oils in an e-cigarette, vape pen, or electronic nicotine product*; (4) *Use marijuana some other way*. Options 1 and 2 were combined into a single binary measure of *smoked cannabis*. Then, a 32-category variable was constructed to assess all possible combinations of past 30-day cigarette smoking, nicotine use with an e-product, cannabis smoking (without an e-product), cannabis use with an e-product, and other form(s) of cannabis use. Participants were assigned a single value ranging from no use to use of cigarettes, use of nicotine with an e-product, and use of cannabis with all 3 methods. Frequencies of use in the past 30 days (ranging from 1 to 30 days) were based on derived variables for both cigarettes and electronic nicotine products. For cannabis, this was determined with the question, *In the past 30 days, on how many days did you use marijuana*?

Control variables included sex, race, age, and household income; lifetime uses of cigarettes, electronic nicotine products, other tobacco products (cigar, cigarillo, filtered cigar, any cigar, pipe, hookah, snus, smokeless, dissolvable tobacco, bidis, and kretek), and marijuana; lifetime diagnoses of high blood pressure, high cholesterol, diabetes, bronchitis, and asthma (adults and youth); lifetime diagnoses of congestive heart failure, stroke, heart attack, other heart conditions, chronic obstructive pulmonary disease, emphysema, and other respiratory conditions; and use of beta blockers (adults only). For lifetime marijuana use and the lifetime cardiovascular and respiratory issues, all relevant lifetime and past 12-month questions from Waves 1 to 6 were used. Appendix Tables E–G (available online) contain prevalence rates for diagnoses of these issues (the footnotes of these Appendix Tables, available online, provide additional coding information). It should be highlighted that this set of control variables are necessary for the adjusted models given that each of these factors is associated with respiratory issues and must be accounted for in the models to determine the independent effect of past 30-day nicotine and cannabis use (Appendix Table N, available online, provides a correlation matrix for each of the variables used in the analyses). Table 1 provides details on how key demographics and substance use variables were coded.

### Statistical Analysis

Two sets of binary logistic regression models were fitted to assess the association between the major independent and dependent variables. First, 31 binary variables (corresponding to the 32 categories of past 30-day substance use, excluding the reference group of no use) were used to predict self-reported respiratory issues among all respondents when controlling for the measures outlined earlier. These 31 binary variables were coded to be mutually exclusive, such that a participant could belong to only 1 substance use group. Second, a set of 31 continuous variables was created to assess how frequency of use was associated with self-reported respiratory symptoms. In this analysis, each participant had a value ranging from 1 to 30 days for a single continuous variable (corresponding to their substance use group) and 0 for the other 30 continuous variables. Only participants who used at least 1 substance (cigarettes, nicotine with an e-product, or cannabis) were included in the models. The maximum number of days across all substances the participant used was taken. The same measures outlined previously were controlled for. Both AOR and 95% CIs are provided on the basis of these analyses. In addition, for the second analysis, unadjusted ORs were calculated by regressing each outcome on a single frequency-of-use variable for every subset of respondents corresponding to a specific substance use group. In addition, all analyses used replicate survey weights to compute unbiased estimates of population parameters that account for the complex sampling design of the PATH (i.e., balanced repeated replication). Sample sizes varied because listwise deletion was used for the analyses. All adjusted models were assessed to determine whether multicollinearity was an issue and found that each model had an average variance inflation factor below 3. It should also be highlighted that additional analyses stratified these models (and sample characteristics) by respondents who were aged 12–17 years and respondents who were aged ≥18 years (Appendix Tables A–D and H–M, available online). Owing to sample size constraints, the authors only include the stratified results as Appendix Material (available online) and focus on the full sample of respondents who are aged ≥12 years (age was controlled for in all of the adjusted models).

## RESULTS

Table 1 shows that 14.5% of respondents indicated past 30-day cigarette smoking, 6.4% indicated using nicotine in an e-product during the past 30 days, 11.1% indicated smoking cannabis during the past 30 days, 4.3% indicated using cannabis in an e-product during the past 30 days, and 3.9% used cannabis in some other way during the past 30 days. It should also be noted that 17.6% of the sample had a respiratory symptom index of 2 or higher; the modal past-year self-reported respiratory symptom indicated by respondents was having a dry cough at night not associated with a cold or chest infection (15.9%).

On the basis of the 32-category variable assessing single and dual nicotine and cannabis use (Table 4), past 30-day cigarette smoking only and past 30-day cannabis smoking only were the most prevalent types of use: 8.1% and 3.6%, respectively (73.6% of respondents did not use any nicotine or cannabis product in the past 30 days). Moreover, the most prevalent past 30-day dual nicotine and cannabis use was cannabis smoking and cigarette smoking (2.4%).

**Table 4:** Estimated Distributions of Past 30-Day Substance Use for Participants Aged ≥12 Years in the PATH, Wave 6 (N=36,168)

Past 30-day substance use (mutually exclusive categories)	*n*	% (95% CI)
No use	22,888	73.61 (72.78, 74.41)
Cigarette smoking only	3,438	8.11 (7.75, 8.49)
Cannabis smoking only	1,714	3.64 (3.37, 3.94)
Nicotine use with e-product only	1,423	2.09 (1.93, 2.26)
Cigarette smoking and cannabis smoking	1,202	2.45 (2.27, 2.64)
Cigarette smoking and nicotine use with e-product	778	1.36 (1.24, 1.49)
Cannabis smoking and cannabis use with e-product	584	1.00 (0.87, 1.16)
Other cannabis use only	549	1.90 (1.65, 2.20)
Nicotine use with e-product and cannabis smoking	520	0.67 (0.60, 0.75)
Cigarette smoking, nicotine use with e-product, and cannabis smoking	439	0.71 (0.62, 0.81)
Nicotine use with e-product, cannabis smoking, and cannabis use with e-product	355	0.43 (0.38, 0.50)
Cannabis use with e-product only	318	0.71 (0.58, 0.87)
Cigarette smoking, nicotine use with e-product, cannabis smoking, and cannabis use with e-product	272	0.38 (0.33, 0.44)
Cigarette smoking, cannabis smoking, and cannabis use with e-product	243	0.45 (0.39, 0.53)
Cannabis smoking and other cannabis use	205	0.44 (0.33, 0.57)
Cannabis smoking, cannabis use with e-product, and other cannabis use	184	0.34 (0.28, 0.43)
Nicotine use with e-product and cannabis use with e-product	127	0.16 (0.13, 0.20)
Cigarette smoking and other cannabis use	114	0.24 (0.19, 0.31)
Cigarette smoking, cannabis smoking, and other cannabis use	84	0.17 (0.13, 0.23)
Nicotine use with e-product, cannabis smoking, cannabis use with e-product, and other cannabis use	79	0.11 (0.08, 0.14)
Cannabis use with e-product and other cannabis use	72	0.21 (0.14, 0.30)
Cigarette smoking and cannabis use with e-product	63	0.14 (0.10, 0.21)
Cigarette smoking, cannabis smoking, cannabis use with e-product, and other cannabis use	62	0.12 (0.09, 0.17)
Cigarette smoking, nicotine use with e-product, cannabis smoking, cannabis use with e-product, and other cannabis use	60	0.09 (0.06, 0.12)
Cigarette smoking, nicotine use with e-product, and cannabis use with e-product	59	0.10 (0.07, 0.15)
Nicotine use with e-product and other cannabis use	51	0.10 (0.06, 0.14)
Nicotine use with e-product, cannabis smoking, and other cannabis use	49	0.09 (0.04, 0.17)
Cigarette smoking, nicotine use with e-product, and other cannabis use	47	0.07 (0.05, 0.10)
Cigarette smoking, nicotine use with e-product, cannabis smoking, and other cannabis use	29	0.04 (0.03, 0.07)
Nicotine use with e-product, cannabis use with e-product, and other cannabis use	17	0.02 (0.01, 0.04)
Cigarette smoking, nicotine use with e-product, cannabis use with e-product, and other cannabis use	10	0.02 (0.01, 0.05)
Cigarette smoking, cannabis use with e-product, and other cannabis use	10	0.02 (0.01, 0.04)

*Notes: n*=unweighted sample size; percentages and 95% CIs incorporate cross-sectional replicate weights (Wave 4 cohort).

PATH, Population Assessment of Tobacco and Health

Table 2, Table 3 provides the results assessing the association between respiratory symptoms and past 30-day single and dual nicotine and cannabis use. Focusing on the 5 most prevalent types of single and dual nicotine and cannabis use (this represents roughly 67% of respondents who indicated using either nicotine or cannabis during the past 30 days), the analysis shows that cigarette smoking and cannabis smoking during the past 30 days were associated with significantly greater odds of indicating each of the past-year respiratory symptoms (when compared with that among those who did not use nicotine or cannabis during the past 30 days). Cigarette smoking only and cigarette smoking and nicotine use with an e-product during the past 30 days were associated with significantly greater odds of indicating 4 of the 5 past-year respiratory symptoms than their nonusing peers. In addition, cannabis smoking only had significantly greater odds of indicating only 1 of the past-year respiratory symptoms than among respondents who did not use any of these substances during the past 30 days. Respondents who indicated nicotine use with an e-product only were not found to have statistically significant greater odds of indicating any of the respiratory symptoms than their peers who did not use nicotine or cannabis during the past 30 days. Moreover, each of these modal forms (5 most prevalent combinations of nicotine or cannabis use) of nicotine and cannabis use during the past 30 days (excluding nicotine use with an e-product only) substantially increased the odds of indicating a respiratory symptom index of 2 or higher when compared with that among the nonusing peers. For instance, the odds of indicating a respiratory symptom index of 2 or higher was roughly 4 times greater (AOR=3.85; 95% CI=3.11, 4.77) among respondents indicating both past 30-day cigarette smoking and cannabis smoking than among their peers who did not use any nicotine or cannabis during the past 30 days.

Table 5, Table 6 provide the results assessing the association between past-year respiratory symptoms and the frequency (number of days used) of past 30-day single and dual nicotine and cannabis use. Highlighting the 5 most prevalent types of single and dual nicotine and cannabis use, the analysis shows that frequency of cigarette smoking and cannabis smoking during the past 30 days was associated with significantly greater odds of indicating each of the past-year respiratory symptoms. Frequency of cigarette smoking only and frequency of cigarette smoking and nicotine use with and e-product during the past 30 days were associated with significantly greater odds of indicating 4 of the 5 past-year respiratory symptoms, whereas frequency of cannabis smoking only had significantly greater odds of indicating 3 of the past-year respiratory symptoms. Frequency of nicotine use with an e-product only were not found to be associated with greater odds of indicating any of the past-year respiratory symptoms. Moreover, the frequency of each of these modal forms of nicotine and cannabis use during the past 30 days (excluding nicotine use with an e-product only) was associated with an increase in the odds of indicating a respiratory symptom index of 2 or higher.

**Table 5:** Past-Year Self-Reported Respiratory Symptoms as a Function of Past 30-Day Substance Use Frequency Among U.S. Participants Aged ≥12 Years

	Wheezing or whistling in the chest		Sleep disturbed owing to wheezing		Speech limited owing to wheezing	
Measure	uOR (95% CI)	AOR (95% CI)	uOR (95% CI)	AOR (95% CI)	uOR (95% CI)	AOR (95% CI)
Past 30-day substance use (mutually exclusive frequency variables: 1-30 days)		*n*=11,439		*n*=11,434		*n*=11,437
Cigarette smoking only	1.05 (1.04, 1.06)	1.04 (1.03, 1.05)	1.03 (1.02, 1.05)	1.03 (1.02, 1.04)	1.03 (1.00, 1.06)	1.00 (0.98, 1.02)
Cannabis smoking only	1.02 (0.99, 1.04)	1.02 (1.00, 1.03)	1.04 (1.01, 1.07)	1.02 (1.00, 1.04)	1.02 (0.99, 1.06)	1.00 (0.97, 1.03)
Nicotine use with e-product only	1.00 (0.98, 1.03)	0.99 (0.97, 1.00)	0.99 (0.95, 1.04)	0.98 (0.96, 1.00)	1.00 (0.94, 1.06)	0.97 (0.93, 1.02)
Cigarette smoking and cannabis smoking	1.07 (1.04, 1.11)	1.04 (1.03, 1.05)	1.04 (0.99, 1.09)	1.03 (1.02, 1.05)	1.16 (0.91, 1.47)	1.02 (1.00, 1.04)
Cigarette smoking and nicotine use with e-product	1.05 (1.01, 1.09)	1.03 (1.01, 1.04)	1.04 (0.97, 1.12)	1.02 (1.01, 1.04)	0.99 (0.92, 1.07)	1.00 (0.97, 1.03)
Cannabis smoking and cannabis use with e-product	1.04 (1.00, 1.07)	1.01 (0.99, 1.03)	1.02 (0.96, 1.08)	1.00 (0.97, 1.03)	1.03 (0.97, 1.10)	1.02 (0.99, 1.05)
Other cannabis use only	1.00 (0.96, 1.04)	1.01 (0.98, 1.05)	1.02 (0.97, 1.07)	1.03 (0.99, 1.07)	1.01 (0.92, 1.10)	1.02 (0.97, 1.08)
Nicotine use with e-product and cannabis smoking	1.01 (0.98, 1.05)	1.01 (0.99, 1.02)	0.95 (0.88, 1.03)	0.97 (0.94, 1.00)	1.02 (0.92, 1.13)	0.97 (0.91, 1.03)
Cigarette smoking, nicotine use with e-product, and cannabis smoking	1.07 (1.02, 1.12)	1.03 (1.02, 1.05)	1.21 (1.06, 1.37)	1.03 (1.01, 1.05)	1.09 (0.96, 1.23)	1.02 (0.99, 1.05)
Nicotine use with e-product, cannabis smoking, and cannabis use with e-product	1.07 (1.00, 1.14)	1.00 (0.98, 1.02)	1.02 (0.94, 1.11)	0.98 (0.95, 1.00)	0.98 (0.91, 1.06)	1.00 (0.96, 1.04)
Cannabis use with e-product only	1.03 (0.97, 1.10)	1.03 (0.98, 1.08)	1.10 (1.00, 1.22)	1.06 (1.00, 1.12)	1.10 (0.95, 1.28)	1.04 (0.96, 1.13)
Cigarette smoking, nicotine use with e-product, cannabis smoking, and cannabis use with e-product	1.04 (0.98, 1.11)	1.05 (1.03, 1.06)	1.05 (0.93, 1.20)	1.05 (1.02, 1.07)	1.00 (0.86, 1.15)	1.03 (0.99, 1.07)
Cigarette smoking, cannabis smoking, and cannabis use with e-product	1.07 (0.98, 1.18)	1.04 (1.02, 1.06)	1.08 (0.94, 1.25)	1.02 (1.00, 1.04)	NA	1.02 (0.98, 1.06)
Cannabis smoking and other cannabis use	1.10 (1.05, 1.15)	1.06 (1.03, 1.08)	1.07 (1.01, 1.13)	1.05 (1.01, 1.09)	1.18 (0.88, 1.58)	1.05 (1.00, 1.10)
Cannabis smoking, cannabis use with e-product, and other cannabis use	1.06 (0.99, 1.13)	1.03 (1.00, 1.06)	1.14 (0.94, 1.37)	1.02 (0.98, 1.05)	1.02 (0.89, 1.17)	1.03 (0.99, 1.07)
Nicotine use with e-product and cannabis use with e-product	1.02 (0.96, 1.07)	1.03 (1.00, 1.06)	0.95 (0.84, 1.08)	0.95 (0.85, 1.05)	1.08 (0.90, 1.30)	1.03 (0.96, 1.12)
Cigarette smoking and other cannabis use	1.07 (0.99, 1.15)	1.06 (1.03, 1.08)	1.12 (1.00, 1.26)	1.05 (1.02, 1.08)	1.06 (0.97, 1.16)	1.03 (0.99, 1.07)
Cigarette smoking, cannabis smoking, and other cannabis use	1.03 (0.97, 1.10)	1.05 (1.02, 1.08)	NA	1.03 (0.99, 1.06)	35.63 (3.21, 395.61)	1.03 (0.98, 1.10)
Nicotine use with e-product, cannabis smoking, cannabis use with e-product, and other cannabis use	1.02 (0.92, 1.12)	1.04 (1.02, 1.07)	0.97 (0.85, 1.10)	1.02 (0.99, 1.06)	1.10 (0.92, 1.31)	1.07 (1.03, 1.11)
Cannabis use with e-product and other cannabis use	1.10 (0.97, 1.25)	1.05 (1.00, 1.10)	1.00 (0.89, 1.14)	1.03 (0.97, 1.10)	1.07 (1.03, 1.11)	1.03 (0.92, 1.14)
Cigarette smoking and cannabis use with e-product	1.40 (1.07, 1.85)	1.07 (1.03, 1.11)	NA	1.02 (0.98, 1.07)	NA	1.03 (0.98, 1.09)
Cigarette smoking, cannabis smoking, cannabis use with e-product, and other cannabis use	1.11 (0.96, 1.27)	1.07 (1.04, 1.10)	1.00 (0.87, 1.15)	1.03 (1.00, 1.07)	0.96 (0.81, 1.14)	1.04 (0.98, 1.10)
Cigarette smoking, nicotine use with e-product, cannabis smoking, cannabis use with e-product, and other cannabis use	1.05 (0.89, 1.23)	1.07 (1.03, 1.10)	NA	1.07 (1.04, 1.10)	NA	0.99 (0.93, 1.06)
Cigarette smoking, nicotine use with e-product, and cannabis use with e-product	1.03 (0.92, 1.16)	1.02 (0.98, 1.06)	0.99 (0.88, 1.11)	1.01 (0.94, 1.09)	0.91 (0.76, 1.09)	0.97 (0.88, 1.08)
Nicotine use with e-product and other cannabis use	1.06 (0.91, 1.24)	1.01 (0.96, 1.05)	1.01 (0.85, 1.20)	1.01 (0.92, 1.10)	NA	1.04 (0.94, 1.14)
Nicotine use with e-product, cannabis smoking, and other cannabis use	1.03 (0.87, 1.22)	1.01 (0.96, 1.06)	18.96 (1.75, 205.53)	1.00 (0.92, 1.09)	17.07 (1.43, 203.85)	0.98 (0.88, 1.08)
Cigarette smoking, nicotine use with e-product, and other cannabis use	1.06 (0.97, 1.15)	1.05 (1.02, 1.09)	1.05 (0.95, 1.17)	1.05 (1.00, 1.10)	1.01 (0.88, 1.15)	1.04 (0.97, 1.11)
Cigarette smoking, nicotine use with e-product, cannabis smoking, and other cannabis use	NA	1.04 (0.99, 1.08)	NA	1.01 (0.96, 1.06)	NA	1.02 (0.94, 1.10)
Nicotine use with e-product, cannabis use with e-product, and other cannabis use	0.93 (0.68, 1.27)	0.98 (0.88, 1.08)	1.00 (1.00, 1.00)	0.02 (0.00, 8.72)	1.00 (1.00, 1.00)	0.02 (0.00, 14.20)
Cigarette smoking, nicotine use with e-product, cannabis use with e-product, and other cannabis use	5.12 (1.33, 19.76)	1.02 (0.90, 1.15)	0.99 (0.99, 0.99)	0.20 (0.03, 1.58)	0.99 (0.99, 0.99)	0.21 (0.02, 2.03)
Cigarette smoking, cannabis use with e-product, and other cannabis use	8.20 (1.74, 38.53)	1.06 (0.99, 1.14)	7.03 (2.12, 23.32)	1.04 (0.93, 1.17)	1.00 (1.00, 1.00)	0.08 (0.00, 6.90)

*Notes:* NA denotes model that could not estimate owing to perfect prediction of the outcome. Unweighted samples sizes are provided. ORs and 95% CIs are weighted to be representative of the U.S. population. All adjusted models control for sex, race, age, and household income; lifetime uses of cigarettes, electronic nicotine products, other tobacco products, and marijuana; lifetime diagnoses of high blood pressure, high cholesterol, diabetes, bronchitis, and asthma (adults and youth); lifetime diagnoses of congestive heart failure, stroke, heart attack, other heart conditions, COPD, emphysema, and other respiratory conditions; and use of beta blockers (adults only). A uOR represents the regression of a single outcome on a single frequency variable (the number of days a participant used the given substances in the past 30 days), and only the participants in that substance use group are included in the sample, that is, 31 separate unadjusted models were fitted for each outcome.

COPD, chronic obstructive pulmonary disease; uOR, unadjusted OR.

**Table 6:** Past-year Self-Reported Respiratory Symptoms as a Function of Past 30-Day Substance Use Frequency Among U.S. Participants Aged ≥12 Years

	Sounded wheezy during or after exercise		Dry cough at night not associated with cold/chest infection		Respiratory symptom index (≥2)	
Measure	uOR (95% CI)	AOR (95% CI)	uOR (95% CI)	AOR (95% CI)	uOR (95% CI)	AOR (95% CI)
Past 30-day substance use (mutually exclusive frequency variables: 1-30 days)		*n*=11,432		*n*=11,436		*n*=11,402
Cigarette smoking only	1.03 (1.02, 1.05)	1.02 (1.01, 1.03)	1.03 (1.02, 1.04)	1.02 (1.01, 1.03)	1.04 (1.03, 1.06)	1.04 (1.03, 1.04)
Cannabis smoking only	1.02 (1.00, 1.04)	1.01 (1.00, 1.02)	1.00 (0.98, 1.02)	1.00 (0.99, 1.01)	1.02 (1.00, 1.04)	1.01 (1.00, 1.02)
Nicotine use with e-product only	1.00 (0.98, 1.02)	1.00 (0.99, 1.01)	1.02 (0.99, 1.04)	0.99 (0.98, 1.00)	1.00 (0.99, 1.02)	0.99 (0.98, 1.00)
Cigarette smoking and cannabis smoking	1.07 (1.03, 1.11)	1.03 (1.02, 1.04)	1.07 (1.02, 1.12)	1.02 (1.01, 1.03)	1.06 (1.03, 1.09)	1.04 (1.03, 1.05)
Cigarette smoking and nicotine use with e-product	1.04 (0.99, 1.09)	1.01 (1.00, 1.03)	1.05 (1.01, 1.09)	1.01 (1.00, 1.03)	1.05 (1.02, 1.09)	1.02 (1.01, 1.03)
Cannabis smoking and cannabis use with e-product	1.03 (1.00, 1.07)	1.02 (1.00, 1.05)	1.03 (1.00, 1.05)	1.01 (0.99, 1.03)	1.03 (1.01, 1.05)	1.02 (1.00, 1.03)
Other cannabis use only	1.02 (0.98, 1.07)	1.02 (0.99, 1.06)	1.00 (0.97, 1.03)	1.02 (1.00, 1.05)	1.01 (0.98, 1.04)	1.02 (0.99, 1.05)
Nicotine use with e-product and cannabis smoking	1.00 (0.97, 1.04)	1.01 (0.99, 1.03)	1.01 (0.98, 1.05)	1.01 (0.99, 1.02)	1.02 (1.00, 1.05)	1.01 (1.00, 1.02)
Cigarette smoking, nicotine use with e-product, and cannabis smoking	1.05 (0.99, 1.11)	1.03 (1.02, 1.05)	1.04 (1.00, 1.09)	1.03 (1.01, 1.04)	1.05 (1.01, 1.09)	1.03 (1.02, 1.05)
Nicotine use with e-product, cannabis smoking, and cannabis use with e-product	1.00 (0.96, 1.05)	0.99 (0.97, 1.01)	1.01 (0.97, 1.04)	1.01 (0.99, 1.02)	1.05 (1.01, 1.09)	1.01 (0.99, 1.02)
Cannabis use with e-product only	1.02 (0.95, 1.09)	1.03 (0.98, 1.08)	0.98 (0.94, 1.03)	1.00 (0.96, 1.04)	1.02 (0.97, 1.07)	1.01 (0.98, 1.05)
Cigarette smoking, nicotine use with e-product, cannabis smoking, and cannabis use with e-product	1.05 (0.96, 1.14)	1.03 (1.02, 1.05)	1.07 (0.98, 1.16)	1.03 (1.02, 1.05)	1.06 (1.00, 1.12)	1.05 (1.03, 1.06)
Cigarette smoking, cannabis smoking, and cannabis use with e-product	1.04 (0.98, 1.11)	1.02 (1.00, 1.04)	1.05 (0.99, 1.11)	1.02 (1.01, 1.04)	1.06 (1.00, 1.13)	1.04 (1.02, 1.05)
Cannabis smoking and other cannabis use	1.04 (0.99, 1.09)	1.03 (1.01, 1.06)	1.07 (1.02, 1.12)	1.03 (1.00, 1.06)	1.08 (1.03, 1.13)	1.04 (1.02, 1.07)
Cannabis smoking, cannabis use with e-product, and other cannabis use	1.06 (0.99, 1.12)	1.04 (1.01, 1.07)	1.04 (0.97, 1.11)	1.01 (0.99, 1.03)	1.05 (0.99, 1.11)	1.04 (1.01, 1.06)
Nicotine use with e-product and cannabis use with e-product	1.01 (0.96, 1.06)	1.03 (1.00, 1.05)	1.05 (1.01, 1.09)	1.03 (1.00, 1.06)	1.02 (0.97, 1.06)	1.02 (1.00, 1.05)
Cigarette smoking and other cannabis use	1.05 (0.98, 1.12)	1.04 (1.02, 1.06)	1.05 (0.99, 1.12)	1.02 (1.00, 1.04)	1.09 (1.02, 1.17)	1.05 (1.03, 1.08)
Cigarette smoking, cannabis smoking, and other cannabis use	1.00 (0.93, 1.09)	1.01 (0.99, 1.03)	1.04 (0.93, 1.16)	1.02 (0.99, 1.04)	1.04 (0.98, 1.11)	1.04 (1.01, 1.07)
Nicotine use with e-product, cannabis smoking, cannabis use with e-product, and other cannabis use	1.10 (0.96, 1.25)	1.03 (1.00, 1.06)	1.01 (0.91, 1.11)	1.03 (1.00, 1.06)	1.03 (0.93, 1.14)	1.03 (1.01, 1.06)
Cannabis use with e-product and other cannabis use	1.01 (0.93, 1.09)	1.02 (0.98, 1.06)	1.01 (0.92, 1.11)	1.04 (1.00, 1.09)	1.01 (0.92, 1.11)	1.04 (0.99, 1.08)
Cigarette smoking and cannabis use with e-product	NA	1.07 (1.02, 1.12)	1.12 (1.01, 1.24)	1.07 (1.03, 1.10)	1.11 (1.01, 1.22)	1.07 (1.03, 1.10)
Cigarette smoking, cannabis smoking, cannabis use with e-product, and other cannabis use	1.09 (0.93, 1.28)	1.07 (1.04, 1.10)	1.04 (0.88, 1.23)	1.02 (0.99, 1.05)	1.07 (0.95, 1.21)	1.07 (1.04, 1.09)
Cigarette smoking, nicotine use with e-product, cannabis smoking, cannabis use with e-product, and other cannabis use	1.04 (0.88, 1.23)	1.05 (1.01, 1.08)	1.06 (0.81, 1.39)	1.02 (0.99, 1.05)	1.03 (0.88, 1.21)	1.06 (1.03, 1.09)
Cigarette smoking, nicotine use with e-product, and cannabis use with e-product	1.00 (0.91, 1.10)	1.02 (0.99, 1.06)	1.04 (0.91, 1.19)	1.00 (0.97, 1.03)	1.09 (0.96, 1.23)	1.03 (1.00, 1.06)
Nicotine use with e-product and other cannabis use	1.02 (0.91, 1.15)	1.00 (0.95, 1.06)	0.97 (0.90, 1.03)	1.01 (0.96, 1.06)	1.02 (0.93, 1.12)	0.99 (0.95, 1.04)
Nicotine use with e-product, cannabis smoking, and other cannabis use	0.96 (0.86, 1.07)	1.02 (0.97, 1.06)	0.97 (0.87, 1.09)	0.99 (0.95, 1.04)	1.00 (0.89, 1.12)	1.02 (0.97, 1.07)
Cigarette smoking, nicotine use with e-product, and other cannabis use	1.00 (0.93, 1.08)	1.02 (0.99, 1.06)	1.05 (0.96, 1.14)	1.03 (1.00, 1.07)	1.05 (0.97, 1.14)	1.04 (1.01, 1.08)
Cigarette smoking, nicotine use with e-product, cannabis smoking, and other cannabis use	NA	1.01 (0.98, 1.05)	1.01 (0.83, 1.22)	1.01 (0.97, 1.06)	1.06 (0.82, 1.36)	1.03 (0.99, 1.07)
Nicotine use with e-product, cannabis use with e-product, and other cannabis use	0.93 (0.68, 1.27)	0.98 (0.88, 1.08)	0.94 (0.86, 1.04)	0.99 (0.89, 1.11)	0.93 (0.68, 1.27)	0.95 (0.85, 1.07)
Cigarette smoking, nicotine use with e-product, cannabis use with e-product, and other cannabis use	0.01 (0.00, 0.01)	0.94 (0.85, 1.05)	0.99 (0.99, 0.99)	0.23 (0.03, 1.65)	5.12 (1.33, 19.76)	1.00 (0.88, 1.13)
Cigarette smoking, cannabis use with e-product, and other cannabis use	7.03 (2.12, 23.32)	1.02 (0.93, 1.12)	6.17 (1.63, 23.34)	0.97 (0.85, 1.10)	8.20 (1.74, 38.53)	1.04 (0.96, 1.12)

*Notes:* NA denotes model that could not estimate owing to perfect prediction of the outcome. Unweighted samples sizes are provided. ORs and 95% CIs are weighted to be representative of the U.S. population. All adjusted models control for sex, race, age, and household income; lifetime uses of cigarettes, electronic nicotine products, other tobacco products, and marijuana; lifetime diagnoses of high blood pressure, high cholesterol, diabetes, bronchitis, and asthma (adults and youth); lifetime diagnoses of congestive heart failure, stroke, heart attack, other heart conditions, COPD, emphysema, and other respiratory conditions; and use of beta blockers (adults only). An uOR represents the regression of a single outcome on a single frequency variable (the number of days a participant used the given substances in the past 30 days), and only the participants in that substance use group are included in the sample, that is, 31 separate unadjusted models were fitted for each outcome.

COPD, chronic obstructive pulmonary disease; uOR, unadjusted OR.

It should also be noted that in supplementary analyses, the results were also stratified into 2 age groups: adolescents (aged 12–17 years) and adults (aged ≥18 years). Appendix Tables C and D (available online) show the prevalence of past 30-day substance use separately for adult (aged ≥18 years) and youth (aged 12–17 years) respondents. Among adults, 73.2% indicated no use of cigarettes, nicotine from e-products, or cannabis, compared with 86.9% of youth. For adult respondents, the 5 most prevalent use categories were (1) cigarette smoking only (8.3%), (2) cannabis smoking only (3.7%), (3) cigarette smoking and cannabis smoking (2.5%), (4) nicotine use with e-product only (2.1%), and (5) other cannabis use only (2.0%). Among youth, the 5 most prevalent categories were (1) nicotine use with e-product only (3.4%); (2) cannabis smoking only (2.0%); (3) nicotine use with e-product, cannabis smoking, and cannabis use with e-product (1.3%); (4) nicotine use with e-product and cannabis smoking (1.1%); and (5) cannabis smoking and cannabis use with e-product (1.0%). Overall, adult and youth respondents shared 2 of the 5 most prevalent use categories. Differences between adults and youth in their most prevalent use categories largely result from deviations in cigarette use and nicotine use with an e-product.

Owing to a limited number of youth respondents, the authors were unable to fit each model separately on this subset of the sample. However, by examining the prevalence of the outcomes by the most common of the 32 substance use categories, the authors generally see agreement between adults and youth in the association of substance use categories with respiratory outcomes. For example, among respondents indicating no substance use, 13.0% of adults had a respiratory symptom index of ≥2, compared with 11.7% of youth. For those indicating nicotine use with an e-product only, 15.1% of adults had a respiratory symptom index of ≥2, compared with 19.3% of youth. For those indicating cannabis smoking only, the prevalence for a respiratory symptom index of ≥2 was 19.5% for adults and 13.7% for youth. Finally, among respondents indicating cannabis smoking and cannabis use with an e-product, the prevalence for a respiratory symptom index of ≥2 was 22.2% for adults and 23.9% for youth. Readers may refer to Appendix Tables H–K (available online) for all outcomes.

## DISCUSSION

Using the most recent estimates available of the U.S. population's use of cigarettes, nicotine with an e-product, and cannabis (with or without an e-product), this study found several key associations with respiratory health symptoms. In particular, past 30-day cigarette smoking only was one of the strongest predictors (on the basis of the AORs) of indicating the majority of the past-year respiratory symptoms assessed in this study. Notably, the combination of past 30-day cigarette smoking and cannabis smoking had a substantial association with indicating any past-year respiratory symptom and a respiratory index of ≥2. This finding builds on previous work and highlights that cigarette smoking only had similar associations with indicating several past-year respiratory symptoms (or a respiratory symptom index of ≥2) when compared with those among respondents who used all of these products (i.e., cigarette smoking, nicotine in an e-product, cannabis smoking, and cannabis in an e-product).^8^ These findings warrant continued efforts to minimize the harmful use of both combustible cigarette and cannabis use within the population given the growing accessibility and use of cannabis within the U.S.

The most common combination of past 30-day nicotine and cannabis use in this U.S. sample was cigarette smoking and cannabis smoking. This represented roughly 1 of 10 (9.3%) individuals who used either nicotine or cannabis during the past 30 days. Importantly, this modal dual-use combination was associated with greater risk for all respiratory symptoms, and nearly 1 half of individuals (42.4%) who reported this pattern had a respiratory index score that indicated likely functional impairment (i.e., a respiratory symptom index of ≥2). Moreover, across all possible patterns of use, the risk of respiratory symptoms (or a respiratory symptom index of ≥2) significantly increased when either nicotine or cannabis was smoked using some form of combustible product (e.g., cigarette or blunt). Given that those smoking both cigarettes and cannabis are more likely to have negative respiratory health, along with having a higher risk for tobacco and cannabis use disorder symptoms, cessation services should be tailored for this type dual-use behavior that involve combustibles.

Although this study found that past 30-day nicotine and cannabis use in an e-product only was not associated with any past respiratory symptoms (or a respiratory index of ≥2), it is important to note that most individuals who used nicotine or cannabis in an e-product also smoked cigarettes or cannabis. Thus, nicotine or cannabis use in an e-product may indirectly place individuals at higher risk for respiratory symptoms through the use of other combustible types of nicotine and cannabis products. Furthermore, individuals who used nicotine or cannabis in an e-product only had a relatively low number of days of use during a 30-day period when compared with the other cigarette and cannabis smokers in the study sample—this also helps to explain the null results with respect to both the frequency of use and respiratory symptoms among nicotine e-product–only users and cannabis e-product–only users.

### Limitations

The authors note several study limitations. For instance, this study can only detect associations given the cross-sectional approach of the study. Although this is a clear limitation, the current analyses captured a robust association between respiratory symptoms and cigarette smoking and cannabis smoking among a recent 2021 national sample that included both adolescent and adults. Another limitation is that co-use of other nicotine products (beyond cigarettes and nicotine use with e-products) was not assessed given the scope of the study; however, other types of past-year tobacco use were controlled for in all of the multivariable models. Future research may explore other combinations to assess whether these substances are putting people at greater risk for respiratory issues because past research has found that co-use is related to respiratory symptoms among adults.^27^

## CONCLUSIONS

Despite these limitations, this study provides robust evidence regarding the potential harms of single or dual nicotine (i.e., cigarette use) and cannabis use through combustible products among a recent 2021 national sample that included both adolescents and adults. Although it has been suggested that respiratory issues may stem from local electronic liquids/additives as they relate to both nicotine and cannabis,^28^ the data from this analysis suggest that the inhalation of nicotine from combustible cigarettes or from smoking cannabis is associated with the greatest pulmonary irritation and symptoms of lung diseases (both known and unknown). Indeed, the respiratory symptoms identified here may be a harbinger of later respiratory problems that deserve closer monitoring.

## Appendix Supplementary materials

Image, application 1

Image, application 2

Image, application 3

Image, application 4

Image, application 5

Image, application 6

Image, application 7

Image, application 8

Image, application 9

Image, application 10

Image, application 11

Image, application 12

Image, application 13

Image, application 14

## Footnotes

Supplementary material associated with this article can be found in the online version at doi:10.1016/j.focus.2024.100291.
