# Supplementary material for: Assessing the Association Between Respiratory Symptoms and Nicotine and Cannabis Use Through Traditional and E-Product Devices in the U.S
Source: AJPM Focus. 2024 Oct 22;4(1):100291. doi: 10.1016/j.focus.2024.100291 (PMC11994035; doi:10.1016/j.focus.2024.100291)
Supplement: Supplementary file 1 [file mmc1.docx]

**Supplemental Table A. Estimated Distributions of Key Study Measures for Participants Ages 18+ in the PATH, Wave 6 (n=30516)**

|  | **n** | **% (95% CI)** |
| --- | --- | --- |
| **Sex^a^** |  |  |
| Male | 14561 | 46.57 (46.09, 47.04) |
| Female | 15916 | 53.43 (52.96, 53.91) |
| **Race^b^** |  |  |
| White | 20739 | 76.73 (76.16, 77.28) |
| Black | 5016 | 12.65 (12.32, 12.99) |
| Other | 3644 | 10.62 (10.20, 11.06) |
| **Age^c^** |  |  |
| 18 to 34 | 17677 | 26.39 (25.89, 26.90) |
| 35 to 54 | 6640 | 32.18 (31.47, 32.90) |
| 55 and older | 6199 | 41.43 (40.73, 42.13) |
| **Household income^d^** |  |  |
| $9,999 or lower | 3343 | 7.21 (6.74, 7.71) |
| $10,000 to $24,999 | 4666 | 13.71 (13.09, 14.35) |
| $25,000 to $49,999 | 6494 | 20.56 (19.77, 21.38) |
| $50,000 to $99,999 | 7530 | 28.33 (27.34, 29.35) |
| $100,000 or higher | 6523 | 27.69 (26.71, 28.69) |
| Nonresponse follow-up: above $50,000 | 494 | 1.38 (1.15, 1.66) |
| Nonresponse follow-up: below $50,000 | 446 | 1.11 (0.95, 1.30) |
| **Lifetime substance use^e^** |  |  |
| Smoked cigarettes | 19013 | 64.73 (63.20, 66.23) |
| Used e-product (nicotine) | 15304 | 29.24 (28.43, 30.07) |
| Used other form(s) of tobacco | 17896 | 50.77 (49.38, 52.16) |
| Used cannabis in any way | 17457 | 46.28 (44.97, 47.60) |
| **Past 30-day substance use^f^** |  |  |
| Smoked cigarettes | 6891 | 14.93 (14.41, 15.47) |
| Used e-product (nicotine) | 4012 | 6.41 (6.15, 6.69) |
| Used cannabis in any way | 7195 | 15.04 (14.38, 15.73) |
| Smoked cannabis | 5819 | 11.26 (10.75, 11.80) |
| Used cannabis in an e-product | 2331 | 4.30 (4.03, 4.59) |
| Used cannabis in some other way | 1571 | 4.05 (3.69, 4.44) |
| **Lifetime self-reported respiratory symptoms** |  |  |
| Wheezing or whistling in chest | 13093 | 38.59 (37.60, 39.58) |
| **Past-year self-reported respiratory symptoms** |  |  |
| Wheezing or whistling in chest | 4074 | 11.93 (11.34, 12.53) |
| Sleep disturbed due to wheezing | 1632 | 4.76 (4.42, 5.12) |
| Speech limited due to wheezing | 682 | 1.88 (1.68, 2.10) |
| Sounded wheezy during or after exercise | 3222 | 8.70 (8.18, 9.26) |
| Dry cough at night not associated with cold / chest infection | 4961 | 16.10 (15.40, 16.83) |
| Respiratory symptom index (2 or more) | 6032 | 17.70 (17.02, 18.40) |

Notes: n = unweighted sample size; percentages and 95% confidence intervals incorporate cross-sectional replicate weights (wave 4 cohort).

^a^Sex of participant was a derived variable, i.e., Population Assessment of Tobacco and Health (PATH) Study constructed the variable from the interview; sex was coded as “Male” or “Female”.

^b^Race of participant was a derived variable from the interview and was coded as “White alone”, “Black alone”, or “Other”.

^c^Age of respondent was a derived variable from the interview.

^d^Household income was a derived variable from the interview and was coded as “Less than $10,000”, “$10,000 to $24,999”, “$25,000 to $49,999”, “$50,000 to $99,999”, or “$100,000 or more”. Participants who did not know or refused to report their household income were probed further to respond either “Above $50,000” or “Below $50,000”.

^e^Lifetime cigarette, e-product (nicotine), and other tobacco use were coded from derived variables provided by the PATH at wave 6. Other tobacco use included the following products: traditional cigar, cigarillo, filtered cigar, pipe, hookah, snus, smokeless tobacco, dissolvable tobacco, IQOS, bidi, and kretek. Lifetime cannabis use was coded from all lifetime and past 12-month wave 1-6 questions asking, “have you smoked part or all of a traditional cigar, cigarillo, or filtered cigar with marijuana in it?” or “have you used marijuana, hash, THC, grass, pot, or weed?”

^f^Past 30-day cigarette and e-product (nicotine) use were coded from derived variables provided by the PATH at wave 6. Past 30-day cannabis use was coded from the question asking, “have you used marijuana in the past 30 days?” (this measure also used the past 12-month and lifetime questions). Participants who used cannabis in the past 30 days were asked which of the four ways they used it: smoked dried herb or flower in a joint, pipe, hookah, or bong; smoked dried herb or flower in a blunt cigar, cigarillo, or filtered cigar; vaped marijuana liquids or oils in an e-cigarette, vape pen, or electronic nicotine product; or used marijuana some other way. The first two options were combined into one measure, “smoked cannabis”.
