# Supplementary material for: Assessing the Association Between Respiratory Symptoms and Nicotine and Cannabis Use Through Traditional and E-Product Devices in the U.S
Source: AJPM Focus. 2024 Oct 22;4(1):100291. doi: 10.1016/j.focus.2024.100291 (PMC11994035; doi:10.1016/j.focus.2024.100291)
Supplement: Supplementary file 2 [file mmc2.docx]

**Supplemental Table B. Estimated Distributions of Key Study Measures for Participants Ages 12-17 in the PATH, Wave 6 (n=5652)**

|  | **n** | **% (95% CI)** |
| --- | --- | --- |
| **Sex^a^** |  |  |
| Male | 2958 | 50.87 (49.75, 51.99) |
| Female | 2674 | 49.13 (48.01, 50.25) |
| **Race^b^** |  |  |
| White | 3594 | 67.82 (66.64, 68.97) |
| Black | 783 | 15.30 (14.41, 16.24) |
| Other | 937 | 16.88 (15.81, 18.00) |
| **Household income^c^** |  |  |
| $9,999 or lower | 324 | 5.07 (4.28, 5.99) |
| $10,000 to $24,999 | 616 | 10.60 (9.53, 11.77) |
| $25,000 to $49,999 | 1085 | 17.68 (16.09, 19.40) |
| $50,000 to $99,999 | 1389 | 24.78 (23.04, 26.62) |
| $100,000 or higher | 1999 | 39.53 (37.23, 41.89) |
| Nonresponse follow-up: above $50,000 | 73 | 1.54 (1.14, 2.08) |
| Nonresponse follow-up: below $50,000 | 56 | 0.79 (0.51, 1.24) |
| **Lifetime substance use^d^** |  |  |
| Smoked cigarettes | 371 | 8.70 (7.61, 9.94) |
| Used e-product (nicotine) | 1030 | 24.63 (22.63, 26.76) |
| Used other form(s) of tobacco | 324 | 8.00 (6.91, 9.25) |
| Used cannabis in any way | 823 | 20.66 (18.86, 22.58) |
| **Past 30-day substance use^e^** |  |  |
| Smoked cigarettes | 63 | 1.56 (1.14, 2.13) |
| Used e-product (nicotine) | 326 | 7.95 (6.86, 9.21) |
| Used cannabis in any way | 352 | 9.15 (8.10, 10.31) |
| Smoked cannabis | 266 | 6.80 (5.93, 7.79) |
| Used cannabis in an e-product | 186 | 4.89 (4.11, 5.81) |
| Used cannabis in some other way | 52 | 1.47 (1.08, 2.00) |
| **Lifetime self-reported respiratory symptoms** |  |  |
| Wheezing or whistling in chest | 1626 | 34.57 (32.78, 36.41) |
| **Past-year self-reported respiratory symptoms** |  |  |
| Wheezing or whistling in chest | 467 | 8.07 (6.98, 9.32) |
| Sleep disturbed due to wheezing | 101 | 1.90 (1.45, 2.49) |
| Speech limited due to wheezing | 95 | 1.53 (1.09, 2.14) |
| Sounded wheezy during or after exercise | 537 | 8.75 (7.62, 10.02) |
| Dry cough at night not associated with cold / chest infection | 648 | 10.48 (9.05, 12.11) |
| Respiratory symptom index (2 or more) | 703 | 12.58 (11.24, 14.07) |

Notes: n = unweighted sample size; percentages and 95% confidence intervals incorporate cross-sectional replicate weights (wave 4 cohort).

^a^Sex of participant was a derived variable, i.e., Population Assessment of Tobacco and Health (PATH) Study constructed the variable from the interview; sex was coded as “Male” or “Female”.

^b^Race of participant was a derived variable from the interview and was coded as “White alone”, “Black alone”, or “Other”.

^c^Household income was a derived variable from the interview and was coded as “Less than $10,000”, “$10,000 to $24,999”, “$25,000 to $49,999”, “$50,000 to $99,999”, or “$100,000 or more”. Participants who did not know or refused to report their household income were probed further to respond either “Above $50,000” or “Below $50,000”.

^d^Lifetime cigarette, e-product (nicotine), and other tobacco use were coded from derived variables provided by the PATH at wave 6. Other tobacco use included the following products: traditional cigar, cigarillo, filtered cigar, pipe, hookah, snus, smokeless tobacco, dissolvable tobacco, IQOS, bidi, and kretek. Lifetime cannabis use was coded from all lifetime and past 12-month wave 1-6 questions asking, “have you smoked part or all of a traditional cigar, cigarillo, or filtered cigar with marijuana in it?” or “have you used marijuana, hash, THC, grass, pot, or weed?”

^e^Past 30-day cigarette and e-product (nicotine) use were coded from derived variables provided by the PATH at wave 6. Past 30-day cannabis use was coded from the question asking, “have you used marijuana in the past 30 days?” (this measure also used the past 12-month and lifetime questions). Participants who used cannabis in the past 30 days were asked which of the four ways they used it: smoked dried herb or flower in a joint, pipe, hookah, or bong; smoked dried herb or flower in a blunt cigar, cigarillo, or filtered cigar; vaped marijuana liquids or oils in an e-cigarette, vape pen, or electronic nicotine product; or used marijuana some other way. The first two options were combined into one measure, “smoked cannabis”.
