# Supplementary material for: Assessing the Association Between Respiratory Symptoms and Nicotine and Cannabis Use Through Traditional and E-Product Devices in the U.S
Source: AJPM Focus. 2024 Oct 22;4(1):100291. doi: 10.1016/j.focus.2024.100291 (PMC11994035; doi:10.1016/j.focus.2024.100291)
Supplement: Supplementary file 3 [file mmc3.docx]

**Supplemental Table C. Estimated Distributions of Past 30-day Substance Use for Participants Ages 18+ in the PATH, Wave 6 (n=30516)**

| **Past 30-day substance use (mutually exclusive categories)** | **n** | **% (95% CI)** |
| --- | --- | --- |
| No use | 17794 | 73.22 (72.38, 74.04) |
| Cigarette smoking only | 3429 | 8.34 (7.97, 8.73) |
| Cannabis smoking only | 1635 | 3.69 (3.41, 4.00) |
| Nicotine use with e-product only | 1269 | 2.05 (1.89, 2.21) |
| Cigarette smoking and cannabis smoking | 1197 | 2.52 (2.33, 2.72) |
| Cigarette smoking and nicotine use with e-product | 758 | 1.39 (1.26, 1.53) |
| Cannabis smoking and cannabis use with e-product | 543 | 1.00 (0.86, 1.17) |
| Other cannabis use only | 536 | 1.95 (1.69, 2.25) |
| Nicotine use with e-product and cannabis smoking | 481 | 0.65 (0.58, 0.74) |
| Cigarette smoking, nicotine use with e-product, and cannabis smoking | 429 | 0.72 (0.63, 0.83) |
| Nicotine use with e-product, cannabis smoking, and cannabis use with e-product | 303 | 0.41 (0.35, 0.47) |
| Cannabis use with e-product only | 280 | 0.71 (0.57, 0.86) |
| Cigarette smoking, nicotine use with e-product, cannabis smoking, and cannabis use with e-product | 262 | 0.38 (0.33, 0.44) |
| Cigarette smoking, cannabis smoking, and cannabis use with e-product | 240 | 0.46 (0.40, 0.55) |
| Cannabis smoking and other cannabis use | 197 | 0.44 (0.34, 0.58) |
| Cannabis smoking, cannabis use with e-product, and other cannabis use | 178 | 0.35 (0.28, 0.44) |
| Cigarette smoking and other cannabis use | 114 | 0.25 (0.20, 0.32) |
| Nicotine use with e-product and cannabis use with e-product | 111 | 0.15 (0.12, 0.19) |
| Cigarette smoking, cannabis smoking, and other cannabis use | 83 | 0.18 (0.14, 0.24) |
| Nicotine use with e-product, cannabis smoking, cannabis use with e-product, and other cannabis use | 69 | 0.10 (0.07, 0.13) |
| Cannabis use with e-product and other cannabis use | 67 | 0.21 (0.14, 0.30) |
| Cigarette smoking and cannabis use with e-product | 63 | 0.15 (0.10, 0.21) |
| Cigarette smoking, cannabis smoking, cannabis use with e-product, and other cannabis use | 62 | 0.13 (0.09, 0.17) |
| Cigarette smoking, nicotine use with e-product, cannabis smoking, cannabis use with e-product, and other cannabis use | 59 | 0.09 (0.06, 0.13) |
| Cigarette smoking, nicotine use with e-product, and cannabis use with e-product | 57 | 0.10 (0.07, 0.15) |
| Nicotine use with e-product, cannabis smoking, and other cannabis use | 48 | 0.09 (0.04, 0.18) |
| Cigarette smoking, nicotine use with e-product, and other cannabis use | 47 | 0.07 (0.05, 0.11) |
| Nicotine use with e-product and other cannabis use | 47 | 0.09 (0.06, 0.14) |
| Cigarette smoking, nicotine use with e-product, cannabis smoking, and other cannabis use | 29 | 0.04 (0.03, 0.07) |
| Nicotine use with e-product, cannabis use with e-product, and other cannabis use | 16 | 0.02 (0.01, 0.04) |
| Cigarette smoking, cannabis use with e-product, and other cannabis use | 10 | 0.02 (0.01, 0.04) |
| Cigarette smoking, nicotine use with e-product, cannabis use with e-product, and other cannabis use | 9 | 0.02 (0.01, 0.05) |

Notes: n = unweighted sample size; percentages and 95% confidence intervals incorporate cross-sectional replicate weights (wave 4 cohort).
