# Supplementary material for: Assessing the Association Between Respiratory Symptoms and Nicotine and Cannabis Use Through Traditional and E-Product Devices in the U.S
Source: AJPM Focus. 2024 Oct 22;4(1):100291. doi: 10.1016/j.focus.2024.100291 (PMC11994035; doi:10.1016/j.focus.2024.100291)
Supplement: Supplementary file 4 [file mmc4.docx]

**Supplemental Table D. Estimated Distributions of Past 30-day Substance Use for Participants Ages 12-17 in the PATH, Wave 6 (n=5652)**

| **Past 30-day substance use (mutually exclusive categories)** | **n** | **% (95% CI)** |
| --- | --- | --- |
| No use | 5094 | 86.89 (85.34, 88.30) |
| Nicotine use with e-product only | 154 | 3.41 (2.70, 4.30) |
| Cannabis smoking only | 79 | 1.98 (1.52, 2.57) |
| Nicotine use with e-product, cannabis smoking, and cannabis use with e-product | 52 | 1.27 (0.92, 1.75) |
| Cannabis smoking and cannabis use with e-product | 41 | 1.02 (0.70, 1.49) |
| Nicotine use with e-product and cannabis smoking | 39 | 1.09 (0.77, 1.55) |
| Cannabis use with e-product only | 38 | 0.96 (0.68, 1.37) |
| Cigarette smoking and nicotine use with e-product | 20 | 0.45 (0.24, 0.86) |
| Nicotine use with e-product and cannabis use with e-product | 16 | 0.43 (0.23, 0.78) |
| Other cannabis use only | 13 | 0.35 (0.18, 0.69) |
| Nicotine use with e-product, cannabis smoking, cannabis use with e-product, and other cannabis use | 10 | 0.31 (0.17, 0.59) |
| Cigarette smoking, nicotine use with e-product, cannabis smoking, and cannabis use with e-product | 10 | 0.33 (0.16, 0.68) |
| Cigarette smoking, nicotine use with e-product, and cannabis smoking | 10 | 0.22 (0.10, 0.49) |
| Cigarette smoking only | 9 | 0.17 (0.06, 0.49) |
| Cannabis smoking and other cannabis use | 8 | 0.17 (0.07, 0.41) |
| Cannabis smoking, cannabis use with e-product, and other cannabis use | 6 | 0.10 (0.03, 0.34) |
| Cannabis use with e-product and other cannabis use | 5 | 0.18 (0.06, 0.54) |
| Cigarette smoking and cannabis smoking | 5 | 0.13 (0.05, 0.35) |
| Nicotine use with e-product and other cannabis use | 4 | 0.17 (0.05, 0.62) |
| Cigarette smoking, cannabis smoking, and cannabis use with e-product | 3 | 0.09 (0.02, 0.38) |
| Cigarette smoking, nicotine use with e-product, and cannabis use with e-product | 2 | 0.08 (0.01, 0.52) |
| Cigarette smoking, nicotine use with e-product, cannabis smoking, cannabis use with e-product, and other cannabis use | 1 | 0.04 (0.00, 0.58) |
| Cigarette smoking, nicotine use with e-product, cannabis use with e-product, and other cannabis use | 1 | 0.03 (0.00, 0.36) |
| Nicotine use with e-product, cannabis use with e-product, and other cannabis use | 1 | 0.05 (0.00, 0.65) |
| Nicotine use with e-product, cannabis smoking, and other cannabis use | 1 | 0.03 (0.00, 0.41) |
| Cigarette smoking, cannabis smoking, and other cannabis use | 1 | 0.02 (0.00, 0.32) |
| Cigarette smoking, cannabis smoking, cannabis use with e-product, and other cannabis use | 0 | 0.00 (0.00, 0.00) |
| Cigarette smoking, cannabis use with e-product, and other cannabis use | 0 | 0.00 (0.00, 0.00) |
| Cigarette smoking, nicotine use with e-product, cannabis smoking, and other cannabis use | 0 | 0.00 (0.00, 0.00) |
| Cigarette smoking, nicotine use with e-product, and other cannabis use | 0 | 0.00 (0.00, 0.00) |
| Cigarette smoking and other cannabis use | 0 | 0.00 (0.00, 0.00) |
| Cigarette smoking and cannabis use with e-product | 0 | 0.00 (0.00, 0.00) |

Notes: n = unweighted sample size; percentages and 95% confidence intervals incorporate cross-sectional replicate weights (wave 4 cohort).
