# Supplementary material for: Assessing the Association Between Respiratory Symptoms and Nicotine and Cannabis Use Through Traditional and E-Product Devices in the U.S
Source: AJPM Focus. 2024 Oct 22;4(1):100291. doi: 10.1016/j.focus.2024.100291 (PMC11994035; doi:10.1016/j.focus.2024.100291)
Supplement: Supplementary file 5 [file mmc5.docx]

**Supplemental Table E. Estimated Distributions of Lifetime Diagnoses of Cardiovascular and Respiratory Issues for Participants Ages 12+ in the PATH, Wave 6 (n=36168)**

| **Lifetime Diagnoses of the Following Cardiovascular and Respiratory Issues** | **n** | **% (95% CI)** |
| --- | --- | --- |
| High blood pressure | 7658 | 35.26 (34.32, 36.21) |
| High cholesterol | 6718 | 32.90 (32.04, 33.78) |
| Congestive heart failure | 596 | 2.78 (2.47, 3.12) |
| Stroke | 592 | 2.50 (2.19, 2.85) |
| Heart attack or needed bypass surgery | 603 | 2.86 (2.52, 3.24) |
| Other heart condition | 2491 | 11.03 (10.45, 11.64) |
| Use of beta blockers | 2577 | 14.15 (13.39, 14.94) |
| Diabetes | 4883 | 21.48 (20.69, 22.30) |
| Chronic obstructive pulmonary disease | 1344 | 4.81 (4.41, 5.25) |
| Bronchitis | 4236 | 8.06 (7.66, 8.48) |
| Emphysema | 592 | 2.05 (1.81, 2.32) |
| Asthma | 7467 | 16.67 (16.10, 17.25) |
| Other respiratory condition | 2015 | 8.54 (8.08, 9.04) |

Notes: n = unweighted sample size; percentages and 95% confidence intervals incorporate cross-sectional replicate weights (wave 4 cohort).

Diagnoses of congestive heart failure, stroke, heart attack, other heart conditions, COPD, emphysema, and other respiratory conditions, and use of beta blockers, were assessed for adults (18+) only. It is assumed that participants ages 12-17 at waves 5 or 6 have not been diagnosed with any of these issues.
