# Supplementary material for: Assessing the Association Between Respiratory Symptoms and Nicotine and Cannabis Use Through Traditional and E-Product Devices in the U.S
Source: AJPM Focus. 2024 Oct 22;4(1):100291. doi: 10.1016/j.focus.2024.100291 (PMC11994035; doi:10.1016/j.focus.2024.100291)
Supplement: Supplementary file 6 [file mmc6.docx]

**Supplemental Table F. Estimated Distributions of Lifetime Diagnoses of Cardiovascular and Respiratory Issues for Participants Ages 18+ in the PATH, Wave 6 (n=30516)**

| **Lifetime Diagnoses of the Following Cardiovascular and Respiratory Issues** | **n** | **% (95% CI)** |
| --- | --- | --- |
| High blood pressure | 7552 | 36.22 (35.26, 37.20) |
| High cholesterol | 6518 | 33.77 (32.88, 34.66) |
| Congestive heart failure | 596 | 2.86 (2.54, 3.22) |
| Stroke | 592 | 2.57 (2.25, 2.93) |
| Heart attack or needed bypass surgery | 603 | 2.94 (2.59, 3.34) |
| Other heart condition | 2491 | 11.36 (10.76, 11.99) |
| Use of beta blockers | 2577 | 14.57 (13.79, 15.38) |
| Diabetes | 4739 | 22.03 (21.22, 22.87) |
| Chronic obstructive pulmonary disease | 1344 | 4.96 (4.54, 5.41) |
| Bronchitis | 3517 | 7.82 (7.41, 8.24) |
| Emphysema | 592 | 2.11 (1.87, 2.39) |
| Asthma | 6333 | 16.48 (15.90, 17.07) |
| Other respiratory condition | 2015 | 8.80 (8.31, 9.30) |

Notes: n = unweighted sample size; percentages and 95% confidence intervals incorporate cross-sectional replicate weights (wave 4 cohort).

Diagnoses of congestive heart failure, stroke, heart attack, other heart conditions, COPD, emphysema, and other respiratory conditions, and use of beta blockers, were assessed for adults (18+) only. It is assumed that participants ages 12-17 at waves 5 or 6 have not been diagnosed with any of these issues.
