# Supplementary material for: Assessing the Association Between Respiratory Symptoms and Nicotine and Cannabis Use Through Traditional and E-Product Devices in the U.S
Source: AJPM Focus. 2024 Oct 22;4(1):100291. doi: 10.1016/j.focus.2024.100291 (PMC11994035; doi:10.1016/j.focus.2024.100291)
Supplement: Supplementary file 7 [file mmc7.docx]

**Supplemental Table G. Estimated Distributions of Lifetime Diagnoses of Cardiovascular and Respiratory Issues for Participants Ages 12-17 in the PATH, Wave 6 (n=5652)**

| **Lifetime Diagnoses of the Following Cardiovascular and Respiratory Issues** | **n** | **% (95% CI)** |
| --- | --- | --- |
| High blood pressure | 106 | 2.47 (1.93, 3.17) |
| High cholesterol | 200 | 3.62 (2.99, 4.37) |
| Diabetes | 144 | 2.84 (2.31, 3.48) |
| Bronchitis | 719 | 16.24 (14.89, 17.68) |
| Asthma | 1134 | 23.17 (21.64, 24.76) |

Notes: n = unweighted sample size; percentages and 95% confidence intervals incorporate cross-sectional replicate weights (wave 4 cohort).

Diagnoses of congestive heart failure, stroke, heart attack, other heart conditions, COPD, emphysema, and other respiratory conditions, and use of beta blockers, were assessed for adults (18+) only. It is assumed that participants ages 12-17 at waves 5 or 6 have not been diagnosed with any of these issues.
