# Supplementary material for: Assessing the Association Between Respiratory Symptoms and Nicotine and Cannabis Use Through Traditional and E-Product Devices in the U.S
Source: AJPM Focus. 2024 Oct 22;4(1):100291. doi: 10.1016/j.focus.2024.100291 (PMC11994035; doi:10.1016/j.focus.2024.100291)
Supplement: Supplementary file 8 [file mmc8.docx]

**Supplemental Table H. Past-year Self-reported Respiratory Symptoms as a Function of Past 30-day Substance Use among U.S. Participants Ages 18+**

|  | **Wheezing or whistling in the chest^a^** | | **Sleep disturbed due to wheezing^b^** | | **Speech limited due to wheezing^c^** | |
| --- | --- | --- | --- | --- | --- | --- |
|  | **%** | **aOR (95% CI)** | **%** | **aOR (95% CI)** | **%** | **aOR (95% CI)** |
| **Past 30-day substance use (mutually exclusive categories)** |  | **n = 27019** |  | **n = 27008** |  | **n = 27016** |
| No use | 8.30 | Reference | 3.10 | Reference | 1.33 | Reference |
| Cigarette smoking only | 27.82 | 3.07 (2.59, 3.63) | 12.10 | 2.44 (1.90, 3.12) | 3.71 | 1.09 (0.76, 1.55) |
| Cannabis smoking only | 13.52 | 1.71 (1.24, 2.35) | 5.28 | 1.55 (0.96, 2.51) | 1.83 | 1.10 (0.60, 2.02) |
| Nicotine use with e-product only | 7.80 | 0.67 (0.48, 0.96) | 3.10 | 0.67 (0.37, 1.21) | 1.18 | 0.52 (0.21, 1.27) |
| Cigarette smoking and cannabis smoking | 31.43 | 3.80 (3.00, 4.81) | 13.74 | 2.85 (2.05, 3.98) | 5.13 | 1.55 (0.98, 2.45) |
| Cigarette smoking and nicotine use with e-product | 21.65 | 1.96 (1.39, 2.77) | 11.39 | 2.09 (1.29, 3.37) | 3.96 | 1.05 (0.46, 2.40) |
| Cannabis smoking and cannabis use with e-product | 11.55 | 1.16 (0.72, 1.87) | 3.80 | 0.93 (0.48, 1.80) | 2.49 | 1.42 (0.73, 2.79) |
| Other cannabis use only | 16.14 | 1.67 (1.09, 2.55) | 7.29 | 2.02 (1.15, 3.53) | 3.56 | 2.34 (0.98, 5.57) |
| Nicotine use with e-product and cannabis smoking | 11.58 | 1.18 (0.76, 1.81) | 2.72 | 0.66 (0.26, 1.68) | 0.79 | 0.34 (0.09, 1.39) |
| Cigarette smoking, nicotine use with e-product, and cannabis smoking | 27.35 | 2.66 (1.78, 3.97) | 12.07 | 1.98 (1.15, 3.42) | 5.49 | 1.54 (0.72, 3.28) |
| Nicotine use with e-product, cannabis smoking, and cannabis use with e-product | 11.35 | 0.97 (0.56, 1.71) | 2.97 | 0.50 (0.24, 1.04) | 1.69 | 0.77 (0.26, 2.26) |
| Cannabis use with e-product only | 12.95 | 1.70 (0.76, 3.79) | 6.41 | 2.50 (0.75, 8.38) | 1.93 | 1.55 (0.33, 7.35) |
| Cigarette smoking, nicotine use with e-product, cannabis smoking, and cannabis use with e-product | 31.65 | 4.32 (2.70, 6.91) | 16.66 | 4.25 (2.12, 8.52) | 5.29 | 2.03 (0.89, 4.64) |
| Cigarette smoking, cannabis smoking, and cannabis use with e-product | 31.06 | 3.12 (1.86, 5.25) | 11.02 | 1.97 (1.07, 3.62) | 4.80 | 1.35 (0.55, 3.32) |
| Cannabis smoking and other cannabis use | 19.63 | 2.23 (1.28, 3.89) | 9.11 | 2.63 (1.19, 5.78) | 2.37 | 1.52 (0.49, 4.67) |
| Cannabis smoking, cannabis use with e-product, and other cannabis use | 20.18 | 2.37 (1.10, 5.14) | 5.12 | 1.36 (0.61, 3.02) | 4.09 | 2.27 (0.92, 5.58) |
| Cigarette smoking and other cannabis use | 41.97 | 4.35 (2.29, 8.27) | 20.47 | 3.55 (1.67, 7.52) | 7.98 | 1.84 (0.77, 4.40) |
| Nicotine use with e-product and cannabis use with e-product | 18.64 | 2.28 (1.08, 4.82) | 1.76 | 0.44 (0.09, 2.12) | 2.83 | 1.56 (0.24, 10.21) |
| Cigarette smoking, cannabis smoking, and other cannabis use | 40.40 | 4.88 (2.24, 10.61) | 14.35 | 2.07 (0.78, 5.49) | 9.59 | 2.17 (0.40, 11.72) |
| Nicotine use with e-product, cannabis smoking, cannabis use with e-product, and other cannabis use | 26.21 | 3.39 (1.61, 7.11) | 9.51 | 1.80 (0.71, 4.55) | 9.23 | 3.62 (0.80, 16.50) |
| Cannabis use with e-product and other cannabis use | 13.51 | 1.95 (0.76, 4.96) | 6.35 | 2.57 (0.87, 7.56) | 1.48 | 1.12 (0.09, 14.79) |
| Cigarette smoking and cannabis use with e-product | 38.78 | 9.12 (2.67, 31.14) | 7.77 | 2.14 (0.58, 7.83) | 3.60 | 2.62 (0.54, 12.75) |
| Cigarette smoking, cannabis smoking, cannabis use with e-product, and other cannabis use | 50.21 | 8.45 (3.78, 18.90) | 15.96 | 3.02 (1.06, 8.56) | 8.12 | 2.74 (0.54, 13.86) |
| Cigarette smoking, nicotine use with e-product, cannabis smoking, cannabis use with e-product, and other cannabis use | 48.51 | 8.19 (2.93, 22.91) | 29.98 | 8.27 (3.04, 22.49) | 3.69 | 0.52 (0.08, 3.38) |
| Cigarette smoking, nicotine use with e-product, and cannabis use with e-product | 18.03 | 1.49 (0.40, 5.59) | 10.75 | 1.36 (0.14, 13.43) | 1.76 | 0.36 (0.02, 6.98) |
| Nicotine use with e-product, cannabis smoking, and other cannabis use | 10.86 | 1.29 (0.46, 3.62) | 2.75 | 0.78 (0.09, 6.93) | 0.62 | 0.29 (0.02, 4.23) |
| Cigarette smoking, nicotine use with e-product, and other cannabis use | 30.72 | 3.99 (1.60, 9.94) | 16.23 | 3.77 (1.00, 14.25) | 7.72 | 2.96 (0.54, 16.17) |
| Nicotine use with e-product and other cannabis use | 10.43 | 1.13 (0.36, 3.51) | 4.80 | 1.38 (0.18, 10.87) | 3.45 | 2.11 (0.16, 27.98) |
| Cigarette smoking, nicotine use with e-product, cannabis smoking, and other cannabis use | 28.43 | 2.31 (0.60, 8.94) | 9.85 | 1.07 (0.25, 4.58) | 5.17 | 0.83 (0.10, 6.76) |
| Nicotine use with e-product, cannabis use with e-product, and other cannabis use | 4.61 | 0.36 (0.02, 7.38) | 0.00 | 0.00 (0.00, 3.67) | 0.00 | 0.00 (0.00, 1.11) |
| Cigarette smoking, cannabis use with e-product, and other cannabis use | 32.78 | 2.70 (0.43, 17.07) | 19.74 | 2.39 (0.18, 32.16) | 0.00 | 0.00 (0.00, 0.38) |
| Cigarette smoking, nicotine use with e-product, cannabis use with e-product, and other cannabis use | 17.55 | 1.44 (0.03, 74.78) | 0.00 | 0.00 (0.00, 2.54) | 0.00 | 0.00 (0.00, 1.01) |

Notes: Unweighted samples sizes are provided. Prevalence, adjusted odds ratios, and 95% confidence intervals are weighted to be representative of the U.S. population. All models control for sex, race, age, and household income; lifetime uses of cigarettes, electronic nicotine products, other tobacco products, and marijuana; lifetime diagnoses of high blood pressure, high cholesterol, diabetes, bronchitis, and asthma (adults and youths); and lifetime diagnoses of congestive heart failure, stroke, heart attack, other heart conditions, COPD, emphysema, and other respiratory conditions, and use of beta blockers (adults only).

^a^This item was measured with the following question: “Have you had wheezing or whistling in the chest in the past 12 months?” Response options were “Yes” and “No”.

^b^This item was measured with the following question: “In the past 12 months, how often, on average has your sleep been disturbed due to wheezing?” Response options were “Never woken with wheezing”, “Less than one night per week”, and “One or more nights per week”. This item was recoded as a binary variable.

^c^This item was measured with the following question: “In the past 12 months, has wheezing ever been severe enough to limit your speech to only one or two words between breaths?” Response options were “Yes” and “No”.
