# Supplementary material for: Assessing the Association Between Respiratory Symptoms and Nicotine and Cannabis Use Through Traditional and E-Product Devices in the U.S
Source: AJPM Focus. 2024 Oct 22;4(1):100291. doi: 10.1016/j.focus.2024.100291 (PMC11994035; doi:10.1016/j.focus.2024.100291)
Supplement: Supplementary file 9 [file mmc9.docx]

**Supplemental Table I. Past-year Self-reported Respiratory Symptoms as a Function of Past 30-day Substance Use among U.S. Participants Ages 18+**

|  | **Sounded wheezy during or after exercise^a^** | | **Dry cough at night not associated with cold / chest infection^b^** | | **Respiratory symptom index (2 or more)** | |
| --- | --- | --- | --- | --- | --- | --- |
|  | **%** | **aOR (95% CI)** | **%** | **aOR (95% CI)** | **%** | **aOR (95% CI)** |
| **Past 30-day substance use (mutually exclusive categories)** |  | **n = 27006** |  | **n = 27010** |  | **n = 26953** |
| No use | 6.23 | Reference | 13.65 | Reference | 13.00 | Reference |
| Cigarette smoking only | 16.39 | 1.92 (1.57, 2.35) | 27.29 | 1.91 (1.65, 2.20) | 36.99 | 3.01 (2.62, 3.46) |
| Cannabis smoking only | 10.13 | 1.33 (0.96, 1.85) | 15.50 | 1.07 (0.82, 1.39) | 19.51 | 1.53 (1.18, 2.00) |
| Nicotine use with e-product only | 9.48 | 1.01 (0.76, 1.35) | 11.61 | 0.81 (0.61, 1.06) | 15.06 | 0.91 (0.69, 1.19) |
| Cigarette smoking and cannabis smoking | 20.50 | 2.40 (1.83, 3.15) | 29.03 | 2.07 (1.67, 2.55) | 42.45 | 3.84 (3.10, 4.77) |
| Cigarette smoking and nicotine use with e-product | 16.53 | 1.46 (1.03, 2.07) | 22.56 | 1.53 (1.18, 1.97) | 30.52 | 1.89 (1.40, 2.56) |
| Cannabis smoking and cannabis use with e-product | 14.38 | 1.69 (1.03, 2.79) | 17.21 | 1.23 (0.82, 1.82) | 22.24 | 1.54 (1.00, 2.38) |
| Other cannabis use only | 12.14 | 1.49 (0.86, 2.59) | 23.10 | 1.66 (1.08, 2.55) | 25.97 | 1.90 (1.28, 2.81) |
| Nicotine use with e-product and cannabis smoking | 11.26 | 1.25 (0.77, 2.00) | 16.44 | 1.30 (0.90, 1.88) | 19.35 | 1.35 (0.91, 2.01) |
| Cigarette smoking, nicotine use with e-product, and cannabis smoking | 23.40 | 2.32 (1.58, 3.42) | 29.79 | 2.15 (1.54, 3.00) | 38.15 | 2.68 (1.84, 3.89) |
| Nicotine use with e-product, cannabis smoking, and cannabis use with e-product | 9.77 | 0.81 (0.48, 1.38) | 17.44 | 1.27 (0.87, 1.84) | 21.01 | 1.29 (0.85, 1.98) |
| Cannabis use with e-product only | 12.91 | 1.83 (0.85, 3.92) | 16.66 | 1.32 (0.77, 2.24) | 18.54 | 1.47 (0.80, 2.70) |
| Cigarette smoking, nicotine use with e-product, cannabis smoking, and cannabis use with e-product | 23.86 | 2.62 (1.60, 4.29) | 32.92 | 2.84 (1.88, 4.29) | 42.51 | 4.30 (2.77, 6.67) |
| Cigarette smoking, cannabis smoking, and cannabis use with e-product | 18.60 | 1.87 (1.09, 3.20) | 27.64 | 1.91 (1.28, 2.86) | 41.82 | 3.19 (1.92, 5.30) |
| Cannabis smoking and other cannabis use | 14.48 | 1.85 (1.09, 3.12) | 18.11 | 1.36 (0.90, 2.06) | 24.86 | 1.83 (1.18, 2.84) |
| Cannabis smoking, cannabis use with e-product, and other cannabis use | 21.16 | 2.66 (1.26, 5.64) | 16.30 | 1.18 (0.71, 1.96) | 31.14 | 2.66 (1.37, 5.18) |
| Cigarette smoking and other cannabis use | 29.74 | 3.09 (1.80, 5.30) | 28.15 | 1.50 (0.85, 2.65) | 51.01 | 3.80 (2.09, 6.92) |
| Nicotine use with e-product and cannabis use with e-product | 16.40 | 1.85 (0.82, 4.19) | 23.85 | 2.02 (1.05, 3.90) | 26.32 | 1.97 (1.01, 3.88) |
| Cigarette smoking, cannabis smoking, and other cannabis use | 19.36 | 1.45 (0.80, 2.60) | 24.47 | 1.51 (0.68, 3.36) | 43.85 | 3.27 (1.51, 7.06) |
| Nicotine use with e-product, cannabis smoking, cannabis use with e-product, and other cannabis use | 18.80 | 1.80 (0.70, 4.65) | 27.14 | 2.19 (1.11, 4.29) | 28.97 | 2.22 (1.07, 4.61) |
| Cannabis use with e-product and other cannabis use | 10.67 | 1.65 (0.77, 3.54) | 23.79 | 2.32 (0.85, 6.32) | 25.65 | 2.95 (1.06, 8.17) |
| Cigarette smoking and cannabis use with e-product | 34.52 | 7.62 (2.04, 28.45) | 47.98 | 6.18 (2.36, 16.18) | 47.01 | 7.67 (2.63, 22.40) |
| Cigarette smoking, cannabis smoking, cannabis use with e-product, and other cannabis use | 45.63 | 8.22 (3.82, 17.71) | 30.93 | 2.09 (0.89, 4.91) | 63.06 | 8.72 (4.33, 17.54) |
| Cigarette smoking, nicotine use with e-product, cannabis smoking, cannabis use with e-product, and other cannabis use | 33.99 | 3.73 (1.23, 11.32) | 31.51 | 1.94 (0.86, 4.37) | 58.28 | 7.05 (3.02, 16.46) |
| Cigarette smoking, nicotine use with e-product, and cannabis use with e-product | 18.41 | 1.82 (0.55, 6.02) | 22.55 | 0.92 (0.31, 2.73) | 32.83 | 2.43 (0.88, 6.69) |
| Nicotine use with e-product, cannabis smoking, and other cannabis use | 14.19 | 2.05 (0.56, 7.50) | 15.38 | 0.99 (0.30, 3.29) | 27.48 | 2.36 (0.71, 7.89) |
| Cigarette smoking, nicotine use with e-product, and other cannabis use | 19.68 | 2.10 (0.76, 5.78) | 27.59 | 2.13 (0.86, 5.28) | 39.07 | 3.30 (1.48, 7.37) |
| Nicotine use with e-product and other cannabis use | 9.14 | 1.05 (0.33, 3.30) | 22.14 | 1.90 (0.45, 8.06) | 14.04 | 0.94 (0.35, 2.56) |
| Cigarette smoking, nicotine use with e-product, cannabis smoking, and other cannabis use | 18.29 | 1.18 (0.45, 3.09) | 25.08 | 1.36 (0.38, 4.92) | 39.59 | 2.31 (0.79, 6.75) |
| Nicotine use with e-product, cannabis use with e-product, and other cannabis use | 4.61 | 0.32 (0.02, 6.45) | 17.81 | 1.42 (0.09, 23.29) | 4.61 | 0.19 (0.01, 4.17) |
| Cigarette smoking, cannabis use with e-product, and other cannabis use | 19.74 | 1.11 (0.12, 10.23) | 7.96 | 0.31 (0.01, 7.05) | 32.78 | 1.44 (0.22, 9.64) |
| Cigarette smoking, nicotine use with e-product, cannabis use with e-product, and other cannabis use | 11.59 | 1.08 (0.03, 38.43) | 0.00 | 0.00 (0.00, 3.86) | 17.55 | 0.88 (0.02, 41.44) |

Notes: Unweighted samples sizes are provided. Prevalence, adjusted odds ratios, and 95% confidence intervals are weighted to be representative of the U.S. population. All models control for sex, race, age, and household income; lifetime uses of cigarettes, electronic nicotine products, other tobacco products, and marijuana; lifetime diagnoses of high blood pressure, high cholesterol, diabetes, bronchitis, and asthma (adults and youths); and lifetime diagnoses of congestive heart failure, stroke, heart attack, other heart conditions, COPD, emphysema, and other respiratory conditions, and use of beta blockers (adults only).

^a^This item was measured with the following question: “In the past 12 months, has your chest sounded wheezy during or after exercise?” Response options were “Yes” and “No”.

^b^This item was measured with the following question: “A dry cough is a cough without phlegm or mucus. In the past 12 months, have you had a dry cough at night?” Response options were “Yes” and “No”.
