# Supplementary material for: Assessing the Association Between Respiratory Symptoms and Nicotine and Cannabis Use Through Traditional and E-Product Devices in the U.S
Source: AJPM Focus. 2024 Oct 22;4(1):100291. doi: 10.1016/j.focus.2024.100291 (PMC11994035; doi:10.1016/j.focus.2024.100291)
Supplement: Supplementary file 10 [file mmc10.docx]

**Supplemental Table J. Past-year Self-reported Respiratory Symptoms as a Function of Past 30-day Substance Use among U.S. Participants Ages 12-17**

|  | **Wheezing or whistling in the chest** | | **Sleep disturbed due to wheezing** | | **Speech limited due to wheezing** | |
| --- | --- | --- | --- | --- | --- | --- |
| **Past 30-day substance use (mutually exclusive categories)** | **n** | **%** | **n** | **%** | **n** | **%** |
| No use | 399 | 7.65 | 84 | 1.85 | 83 | 1.48 |
| Nicotine use with e-product only | 15 | 11.14 | 3 | 3.04 | 2 | 2.78 |
| Cannabis smoking only | 8 | 6.64 | 3 | 0.75 | 1 | 1.81 |
| Nicotine use with e-product, cannabis smoking, and cannabis use with e-product | 5 | 7.45 | 1 | 0.00 | 3 | 2.47 |
| Cannabis smoking and cannabis use with e-product | 8 | 15.86 | 3 | 5.75 | 1 | 2.24 |
| Nicotine use with e-product and cannabis smoking | 4 | 10.74 | 0 | 0.00 | 0 | 0.00 |
| Cannabis use with e-product only | 2 | 7.80 | 0 | 0.00 | 0 | 0.00 |
| Cigarette smoking and nicotine use with e-product | 6 | 5.74 | 1 | 0.00 | 2 | 0.00 |
| Nicotine use with e-product and cannabis use with e-product | 0 | 0.00 | 0 | 0.00 | 0 | 0.00 |
| Other cannabis use only | 3 | 9.72 | 1 | 0.00 | 1 | 0.00 |
| Nicotine use with e-product, cannabis smoking, cannabis use with e-product, and other cannabis use | 2 | 14.95 | 1 | 9.36 | 1 | 9.36 |
| Cigarette smoking, nicotine use with e-product, cannabis smoking, and cannabis use with e-product | 2 | 26.93 | 0 | 0.00 | 0 | 0.00 |
| Cigarette smoking, nicotine use with e-product, and cannabis smoking | 2 | 14.08 | 1 | 14.08 | 0 | 0.00 |
| Cigarette smoking only | 0 | 0.00 | 0 | 0.00 | 0 | 0.00 |
| Cannabis smoking and other cannabis use | 1 | 12.67 | 1 | 12.67 | 0 | 0.00 |
| Cannabis smoking, cannabis use with e-product, and other cannabis use | 1 | 12.19 | 0 | 0.00 | 0 | 0.00 |
| Cannabis use with e-product and other cannabis use | 0 | 0.00 | 0 | 0.00 | 0 | 0.00 |
| Cigarette smoking and cannabis smoking | 0 | 0.00 | 0 | 0.00 | 0 | 0.00 |
| Nicotine use with e-product and other cannabis use | 0 | 0.00 | 0 | 0.00 | 0 | 0.00 |
| Cigarette smoking, cannabis smoking, and cannabis use with e-product | 1 | 30.60 | 0 | 0.00 | 0 | 0.00 |
| Cigarette smoking, nicotine use with e-product, and cannabis use with e-product | 1 | 46.78 | 0 | 0.00 | 0 | 0.00 |
| Cigarette smoking, nicotine use with e-product, cannabis smoking, cannabis use with e-product, and other cannabis use | 1 | 100.00 | 0 | 0.00 | 0 | 0.00 |
| Cigarette smoking, nicotine use with e-product, cannabis use with e-product, and other cannabis use | 0 | 0.00 | 0 | 0.00 | 0 | 0.00 |
| Nicotine use with e-product, cannabis use with e-product, and other cannabis use | 1 | 100.00 | 0 | 0.00 | 0 | 0.00 |
| Nicotine use with e-product, cannabis smoking, and other cannabis use | 0 | 0.00 | 0 | 0.00 | 0 | 0.00 |
| Cigarette smoking, cannabis smoking, and other cannabis use | 0 | 0.00 | 0 | 0.00 | 0 | 0.00 |

Notes: n = number of participants in that substance use group who indicated experiencing the given respiratory symptom in the past year; percentages are weighted to be representative of the U.S. population. Substance use groups containing 0 participants are not shown in this table.
