# Supplementary material for: Assessing the Association Between Respiratory Symptoms and Nicotine and Cannabis Use Through Traditional and E-Product Devices in the U.S
Source: AJPM Focus. 2024 Oct 22;4(1):100291. doi: 10.1016/j.focus.2024.100291 (PMC11994035; doi:10.1016/j.focus.2024.100291)
Supplement: Supplementary file 11 [file mmc11.docx]

**Supplemental Table K. Past-year Self-reported Respiratory Symptoms as a Function of Past 30-day Substance Use among U.S. Participants Ages 12-17**

|  | **Sounded wheezy during or after exercise** | | **Dry cough at night not associated with cold / chest infection** | | **Respiratory symptom index (2 or more)** | |
| --- | --- | --- | --- | --- | --- | --- |
| **Past 30-day substance use (mutually exclusive categories)** | **n** | **%** | **n** | **%** | **n** | **%** |
| No use | 460 | 8.16 | 554 | 9.78 | 596 | 11.72 |
| Nicotine use with e-product only | 21 | 13.57 | 27 | 16.57 | 32 | 19.26 |
| Cannabis smoking only | 7 | 8.32 | 13 | 12.58 | 14 | 13.66 |
| Nicotine use with e-product, cannabis smoking, and cannabis use with e-product | 5 | 7.45 | 12 | 26.22 | 6 | 10.77 |
| Cannabis smoking and cannabis use with e-product | 9 | 18.45 | 8 | 15.83 | 11 | 23.99 |
| Nicotine use with e-product and cannabis smoking | 3 | 7.42 | 10 | 29.32 | 8 | 27.19 |
| Cannabis use with e-product only | 4 | 11.19 | 2 | 0.00 | 2 | 7.80 |
| Cigarette smoking and nicotine use with e-product | 6 | 21.25 | 1 | 0.00 | 8 | 26.99 |
| Nicotine use with e-product and cannabis use with e-product | 1 | 9.04 | 1 | 7.08 | 1 | 9.04 |
| Other cannabis use only | 3 | 9.72 | 2 | 0.00 | 3 | 9.72 |
| Nicotine use with e-product, cannabis smoking, cannabis use with e-product, and other cannabis use | 3 | 28.91 | 2 | 32.14 | 5 | 57.93 |
| Cigarette smoking, nicotine use with e-product, cannabis smoking, and cannabis use with e-product | 2 | 13.17 | 3 | 22.26 | 3 | 28.93 |
| Cigarette smoking, nicotine use with e-product, and cannabis smoking | 2 | 29.56 | 3 | 25.41 | 3 | 29.56 |
| Cigarette smoking only | 1 | 0.00 | 0 | 0.00 | 0 | 0.00 |
| Cannabis smoking and other cannabis use | 1 | 12.67 | 1 | 12.67 | 1 | 12.67 |
| Cannabis smoking, cannabis use with e-product, and other cannabis use | 0 | 0.00 | 0 | 0.00 | 1 | 12.19 |
| Cannabis use with e-product and other cannabis use | 1 | 29.03 | 1 | 14.03 | 0 | 0.00 |
| Cigarette smoking and cannabis smoking | 0 | 0.00 | 0 | 0.00 | 0 | 0.00 |
| Nicotine use with e-product and other cannabis use | 0 | 0.00 | 0 | 0.00 | 0 | 0.00 |
| Cigarette smoking, cannabis smoking, and cannabis use with e-product | 1 | 30.60 | 1 | 30.60 | 1 | 30.60 |
| Cigarette smoking, nicotine use with e-product, and cannabis use with e-product | 1 | 46.78 | 0 | 0.00 | 1 | 46.78 |
| Cigarette smoking, nicotine use with e-product, cannabis smoking, cannabis use with e-product, and other cannabis use | 0 | 0.00 | 0 | 0.00 | 1 | 100.00 |
| Cigarette smoking, nicotine use with e-product, cannabis use with e-product, and other cannabis use | 0 | 0.00 | 0 | 0.00 | 0 | 0.00 |
| Nicotine use with e-product, cannabis use with e-product, and other cannabis use | 1 | 100.00 | 0 | 0.00 | 1 | 100.00 |
| Nicotine use with e-product, cannabis smoking, and other cannabis use | 0 | 0.00 | 1 | 100.00 | 0 | 0.00 |
| Cigarette smoking, cannabis smoking, and other cannabis use | 0 | 0.00 | 0 | 0.00 | 0 | 0.00 |

Notes: n = number of participants in that substance use group who indicated experiencing the given respiratory symptom in the past year; percentages are weighted to be representative of the U.S. population. Substance use groups containing 0 participants are not shown in this table.
