# Supplementary material for: Assessing the Association Between Respiratory Symptoms and Nicotine and Cannabis Use Through Traditional and E-Product Devices in the U.S
Source: AJPM Focus. 2024 Oct 22;4(1):100291. doi: 10.1016/j.focus.2024.100291 (PMC11994035; doi:10.1016/j.focus.2024.100291)
Supplement: Supplementary file 13 [file mmc13.docx]

**Supplemental Table M. Past-year Self-reported Respiratory Symptoms as a Function of Past 30-day Substance Use Frequency among U.S. Participants Ages 18+**

|  | **Sounded wheezy during or after exercise** | | **Dry cough at night not associated with cold / chest infection** | | **Respiratory symptom index (2 or more)** | |
| --- | --- | --- | --- | --- | --- | --- |
|  | **uOR (95% CI)** | **aOR (95% CI)** | **uOR (95% CI)** | **aOR (95% CI)** | **uOR (95% CI)** | **aOR (95% CI)** |
| **Past 30-day substance use (mutually exclusive frequency variables: 1-30 days)** |  | **n = 11066** |  | **n = 11071** |  | **n = 11037** |
| Cigarette smoking only | 1.03 (1.02, 1.05) | 1.02 (1.01, 1.03) | 1.03 (1.02, 1.04) | 1.02 (1.01, 1.03) | 1.04 (1.03, 1.06) | 1.04 (1.03, 1.05) |
| Cannabis smoking only | 1.02 (1.00, 1.04) | 1.01 (1.00, 1.02) | 1.00 (0.98, 1.02) | 1.00 (0.99, 1.01) | 1.02 (1.00, 1.04) | 1.01 (1.00, 1.02) |
| Nicotine use with e-product only | 1.00 (0.98, 1.03) | 1.00 (0.99, 1.01) | 1.02 (1.00, 1.04) | 0.99 (0.98, 1.00) | 1.01 (0.99, 1.02) | 0.99 (0.98, 1.00) |
| Cigarette smoking and cannabis smoking | 1.07 (1.03, 1.11) | 1.03 (1.02, 1.04) | 1.07 (1.02, 1.12) | 1.02 (1.01, 1.03) | 1.06 (1.03, 1.09) | 1.04 (1.03, 1.05) |
| Cigarette smoking and nicotine use with e-product | 1.04 (0.99, 1.08) | 1.01 (1.00, 1.03) | 1.05 (1.01, 1.09) | 1.02 (1.00, 1.03) | 1.05 (1.02, 1.09) | 1.02 (1.01, 1.03) |
| Cannabis smoking and cannabis use with e-product | 1.03 (1.00, 1.07) | 1.02 (1.00, 1.05) | 1.03 (1.00, 1.06) | 1.01 (0.99, 1.03) | 1.03 (1.01, 1.06) | 1.02 (1.00, 1.03) |
| Other cannabis use only | 1.02 (0.97, 1.07) | 1.02 (0.98, 1.06) | 1.00 (0.97, 1.03) | 1.02 (1.00, 1.05) | 1.01 (0.98, 1.04) | 1.02 (0.99, 1.05) |
| Nicotine use with e-product and cannabis smoking | 1.00 (0.96, 1.04) | 1.01 (0.99, 1.03) | 1.02 (0.99, 1.06) | 1.01 (0.99, 1.02) | 1.03 (1.00, 1.06) | 1.01 (1.00, 1.03) |
| Cigarette smoking, nicotine use with e-product, and cannabis smoking | 1.05 (0.99, 1.11) | 1.03 (1.02, 1.05) | 1.04 (1.00, 1.09) | 1.03 (1.01, 1.04) | 1.05 (1.01, 1.09) | 1.03 (1.02, 1.05) |
| Nicotine use with e-product, cannabis smoking, and cannabis use with e-product | 0.99 (0.94, 1.04) | 0.99 (0.97, 1.01) | 1.01 (0.97, 1.05) | 1.01 (0.99, 1.02) | 1.04 (0.99, 1.08) | 1.01 (0.99, 1.02) |
| Cannabis use with e-product only | 1.02 (0.95, 1.10) | 1.03 (0.98, 1.08) | 0.98 (0.94, 1.03) | 1.00 (0.96, 1.04) | 1.02 (0.97, 1.07) | 1.01 (0.98, 1.05) |
| Cigarette smoking, nicotine use with e-product, cannabis smoking, and cannabis use with e-product | 1.04 (0.96, 1.13) | 1.03 (1.02, 1.05) | 1.06 (0.98, 1.16) | 1.03 (1.02, 1.05) | 1.07 (1.00, 1.14) | 1.05 (1.03, 1.06) |
| Cigarette smoking, cannabis smoking, and cannabis use with e-product | 1.04 (0.98, 1.11) | 1.02 (1.00, 1.04) | 1.05 (0.99, 1.11) | 1.02 (1.01, 1.04) | 1.06 (1.00, 1.13) | 1.04 (1.02, 1.05) |
| Cannabis smoking and other cannabis use | 1.04 (0.99, 1.09) | 1.03 (1.00, 1.06) | 1.07 (1.02, 1.11) | 1.03 (1.00, 1.06) | 1.07 (1.02, 1.13) | 1.04 (1.02, 1.07) |
| Cannabis smoking, cannabis use with e-product, and other cannabis use | 1.05 (0.99, 1.12) | 1.04 (1.01, 1.07) | 1.04 (0.97, 1.11) | 1.01 (0.99, 1.03) | 1.05 (0.99, 1.11) | 1.04 (1.01, 1.06) |
| Cigarette smoking and other cannabis use | 1.05 (0.98, 1.12) | 1.04 (1.02, 1.06) | 1.05 (0.99, 1.12) | 1.02 (1.00, 1.04) | 1.09 (1.02, 1.17) | 1.05 (1.03, 1.08) |
| Nicotine use with e-product and cannabis use with e-product | 1.00 (0.95, 1.05) | 1.02 (0.99, 1.06) | 1.05 (1.01, 1.09) | 1.03 (1.01, 1.06) | 1.01 (0.97, 1.06) | 1.02 (1.00, 1.05) |
| Cigarette smoking, cannabis smoking, and other cannabis use | 1.00 (0.93, 1.09) | 1.01 (0.99, 1.03) | 1.04 (0.93, 1.16) | 1.02 (0.99, 1.04) | 1.04 (0.98, 1.11) | 1.04 (1.01, 1.07) |
| Nicotine use with e-product, cannabis smoking, cannabis use with e-product, and other cannabis use | 1.09 (0.96, 1.24) | 1.03 (1.00, 1.06) | 1.00 (0.90, 1.11) | 1.03 (1.00, 1.06) | 1.02 (0.93, 1.13) | 1.03 (1.00, 1.05) |
| Cannabis use with e-product and other cannabis use | 1.01 (0.93, 1.09) | 1.02 (0.98, 1.06) | 1.01 (0.92, 1.12) | 1.04 (1.00, 1.09) | 1.01 (0.92, 1.11) | 1.04 (0.99, 1.08) |
| Cigarette smoking and cannabis use with e-product | 1684.55 (21.10, 134470.73) | 1.07 (1.02, 1.12) | 1.12 (1.01, 1.24) | 1.07 (1.03, 1.10) | 1.11 (1.01, 1.22) | 1.06 (1.03, 1.10) |
| Cigarette smoking, cannabis smoking, cannabis use with e-product, and other cannabis use | 1.09 (0.93, 1.28) | 1.07 (1.04, 1.10) | 1.04 (0.88, 1.23) | 1.02 (0.99, 1.05) | 1.07 (0.95, 1.21) | 1.07 (1.04, 1.09) |
| Cigarette smoking, nicotine use with e-product, cannabis smoking, cannabis use with e-product, and other cannabis use | 1.01 (0.85, 1.21) | 1.05 (1.01, 1.08) | 1.04 (0.79, 1.37) | 1.02 (0.99, 1.05) | 1.09 (0.87, 1.35) | 1.06 (1.03, 1.09) |
| Cigarette smoking, nicotine use with e-product, and cannabis use with e-product | 1.00 (0.90, 1.10) | 1.02 (0.98, 1.06) | 1.04 (0.91, 1.19) | 1.00 (0.97, 1.03) | 1.08 (0.96, 1.23) | 1.03 (1.00, 1.06) |
| Nicotine use with e-product, cannabis smoking, and other cannabis use | 0.96 (0.86, 1.08) | 1.02 (0.97, 1.06) | 0.97 (0.87, 1.08) | 0.99 (0.94, 1.03) | 1.00 (0.89, 1.13) | 1.02 (0.97, 1.07) |
| Cigarette smoking, nicotine use with e-product, and other cannabis use | 1.00 (0.93, 1.08) | 1.02 (0.99, 1.06) | 1.05 (0.96, 1.14) | 1.03 (0.99, 1.07) | 1.05 (0.97, 1.14) | 1.04 (1.01, 1.08) |
| Nicotine use with e-product and other cannabis use | 1.02 (0.90, 1.15) | 1.00 (0.95, 1.06) | 0.96 (0.88, 1.04) | 1.01 (0.96, 1.06) | 1.02 (0.92, 1.12) | 0.99 (0.96, 1.04) |
| Cigarette smoking, nicotine use with e-product, cannabis smoking, and other cannabis use | 6938.98 (54.77, 879111.05) | 1.01 (0.98, 1.05) | 1.01 (0.83, 1.22) | 1.01 (0.97, 1.06) | 1.06 (0.82, 1.36) | 1.03 (0.99, 1.07) |
| Nicotine use with e-product, cannabis use with e-product, and other cannabis use | 27.30 (2.07, 359.99) | 0.97 (0.86, 1.09) | 0.92 (0.81, 1.04) | 0.99 (0.89, 1.11) | 27.30 (2.07, 359.99) | 0.95 (0.84, 1.08) |
| Cigarette smoking, cannabis use with e-product, and other cannabis use | 7.03 (2.12, 23.32) | 1.02 (0.93, 1.12) | 6.17 (1.63, 23.34) | 0.97 (0.85, 1.10) | 8.20 (1.74, 38.53) | 1.04 (0.96, 1.12) |
| Cigarette smoking, nicotine use with e-product, cannabis use with e-product, and other cannabis use | 0.01 (0.00, 0.01) | 0.95 (0.85, 1.06) | 0.99 (0.99, 0.99) | 0.23 (0.03, 1.62) | 5.15 (1.27, 20.78) | 1.00 (0.88, 1.14) |

Notes: Unweighted samples sizes are provided. Odds ratios and 95% confidence intervals are weighted to be representative of the U.S. population. All adjusted models control for sex, race, age, and household income; lifetime uses of cigarettes, electronic nicotine products, other tobacco products, and marijuana; lifetime diagnoses of high blood pressure, high cholesterol, diabetes, bronchitis, and asthma (adults and youths); and lifetime diagnoses of congestive heart failure, stroke, heart attack, other heart conditions, COPD, emphysema, and other respiratory conditions, and use of beta blockers (adults only). An “unadjusted” odds ratio represents the regression of a single outcome on a single frequency variable (the number of days a participant used the given substances in the past 30 days), and only the participants in that substance use group are included in the sample, i.e., 31 separate unadjusted models were fitted for each outcome.
