# Supplementary material for: Assessing the Association Between Respiratory Symptoms and Nicotine and Cannabis Use Through Traditional and E-Product Devices in the U.S
Source: AJPM Focus. 2024 Oct 22;4(1):100291. doi: 10.1016/j.focus.2024.100291 (PMC11994035; doi:10.1016/j.focus.2024.100291)
Supplement: Supplementary file 14 [file mmc14.docx]

**Supplemental Table N. Correlation Matrix for Independent and Dependent Variables for Participants Ages 12+ in the PATH, Wave 6 (n=29153)**

|  | Female | Black | Other Race | 18-34 | 35-54 | 55+ | $10k - 25k | $25k - 50k | $50k - 100k | > $100k | Probe: > $50k | Probe: < $50k | Cigs / Life | Vape / Life | Other Tobacco / Life | Marijuana / Life | High Blood Pressure | High Cholesterol | Heart Failure | Stroke | Heart Attack | Other Heart Cond. | Beta Blockers | Diabetes |
| --- | --- | --- | --- | --- | --- | --- | --- | --- | --- | --- | --- | --- | --- | --- | --- | --- | --- | --- | --- | --- | --- | --- | --- | --- |
| Female | 1.000 | 0.024 | -0.005 | 0.000 | 0.021 | 0.001 | 0.053 | 0.012 | -0.021 | -0.069 | -0.010 | 0.015 | -0.032 | -0.002 | -0.160 | -0.029 | -0.041 | -0.007 | -0.007 | -0.005 | -0.045 | 0.012 | -0.014 | 0.025 |
| Black | 0.024 | 1.000 | -0.169 | 0.015 | -0.015 | 0.003 | 0.086 | 0.031 | -0.061 | -0.140 | -0.022 | 0.014 | -0.067 | -0.060 | -0.010 | 0.003 | 0.075 | -0.017 | 0.036 | 0.027 | 0.008 | -0.010 | 0.035 | 0.056 |
| Other Race | -0.005 | -0.169 | 1.000 | 0.072 | -0.028 | -0.099 | 0.000 | -0.002 | -0.009 | 0.004 | 0.000 | 0.003 | -0.066 | 0.000 | -0.037 | -0.013 | -0.062 | -0.038 | -0.022 | -0.012 | -0.027 | -0.026 | -0.050 | -0.012 |
| 18-34 | 0.000 | 0.015 | 0.072 | 1.000 | -0.513 | -0.496 | 0.001 | 0.023 | -0.001 | -0.082 | 0.052 | 0.019 | -0.228 | 0.170 | -0.011 | 0.071 | -0.329 | -0.343 | -0.125 | -0.119 | -0.121 | -0.136 | -0.246 | -0.238 |
| 35-54 | 0.021 | -0.015 | -0.028 | -0.513 | 1.000 | -0.247 | -0.043 | -0.026 | 0.011 | 0.081 | -0.042 | -0.026 | 0.256 | 0.044 | 0.191 | 0.097 | 0.101 | 0.093 | -0.001 | 0.002 | -0.012 | 0.021 | 0.021 | 0.077 |
| 55+ | 0.001 | 0.003 | -0.099 | -0.496 | -0.247 | 1.000 | 0.068 | 0.011 | -0.003 | -0.049 | -0.020 | 0.012 | 0.265 | -0.150 | 0.043 | -0.028 | 0.431 | 0.439 | 0.192 | 0.182 | 0.198 | 0.221 | 0.362 | 0.302 |
| $10k - 25k | 0.053 | 0.086 | 0.000 | 0.001 | -0.043 | 0.068 | 1.000 | -0.219 | -0.248 | -0.239 | -0.055 | -0.049 | 0.033 | 0.038 | -0.010 | 0.005 | 0.071 | 0.048 | 0.076 | 0.056 | 0.045 | 0.052 | 0.054 | 0.068 |
| $25k - 50k | 0.012 | 0.031 | -0.002 | 0.023 | -0.026 | 0.011 | -0.219 | 1.000 | -0.308 | -0.296 | -0.068 | -0.061 | 0.034 | 0.043 | 0.007 | 0.018 | 0.025 | 0.006 | 0.000 | 0.008 | 0.006 | 0.006 | 0.028 | 0.031 |
| $50k - 100k | -0.021 | -0.061 | -0.009 | -0.001 | 0.011 | -0.003 | -0.248 | -0.308 | 1.000 | -0.336 | -0.077 | -0.069 | 0.008 | 0.003 | 0.026 | 0.009 | -0.016 | -0.010 | -0.032 | -0.041 | -0.023 | -0.011 | -0.015 | -0.022 |
| > $100k | -0.069 | -0.140 | 0.004 | -0.082 | 0.081 | -0.049 | -0.239 | -0.296 | -0.336 | 1.000 | -0.074 | -0.067 | -0.060 | -0.094 | -0.002 | -0.025 | -0.081 | -0.024 | -0.055 | -0.047 | -0.047 | -0.050 | -0.062 | -0.092 |
| Probe: > $50k | -0.010 | -0.022 | 0.000 | 0.052 | -0.042 | -0.020 | -0.055 | -0.068 | -0.077 | -0.074 | 1.000 | -0.015 | -0.065 | -0.021 | -0.055 | -0.034 | -0.034 | -0.025 | -0.011 | -0.013 | -0.014 | -0.019 | -0.025 | -0.027 |
| Probe: < $50k | 0.015 | 0.014 | 0.003 | 0.019 | -0.026 | 0.012 | -0.049 | -0.061 | -0.069 | -0.067 | -0.015 | 1.000 | -0.011 | 0.000 | -0.023 | -0.014 | 0.006 | 0.010 | 0.009 | 0.002 | 0.011 | 0.014 | 0.013 | 0.021 |
| Cigs / Life | -0.032 | -0.067 | -0.066 | -0.228 | 0.256 | 0.265 | 0.033 | 0.034 | 0.008 | -0.060 | -0.065 | -0.011 | 1.000 | 0.400 | 0.568 | 0.437 | 0.250 | 0.214 | 0.086 | 0.082 | 0.089 | 0.129 | 0.163 | 0.167 |
| Vape / Life | -0.002 | -0.060 | 0.000 | 0.170 | 0.044 | -0.150 | 0.038 | 0.043 | 0.003 | -0.094 | -0.021 | 0.000 | 0.400 | 1.000 | 0.415 | 0.444 | -0.003 | -0.050 | 0.001 | 0.013 | 0.002 | 0.016 | -0.028 | -0.006 |
| Other Tobacco / Life | -0.160 | -0.010 | -0.037 | -0.011 | 0.191 | 0.043 | -0.010 | 0.007 | 0.026 | -0.002 | -0.055 | -0.023 | 0.568 | 0.415 | 1.000 | 0.493 | 0.133 | 0.082 | 0.029 | 0.041 | 0.053 | 0.072 | 0.060 | 0.059 |
| Marijuana / Life | -0.029 | 0.003 | -0.013 | 0.071 | 0.097 | -0.028 | 0.005 | 0.018 | 0.009 | -0.025 | -0.034 | -0.014 | 0.437 | 0.444 | 0.493 | 1.000 | 0.054 | 0.024 | -0.004 | 0.010 | 0.013 | 0.049 | 0.008 | 0.026 |
| High Blood Pressure | -0.041 | 0.075 | -0.062 | -0.329 | 0.101 | 0.431 | 0.071 | 0.025 | -0.016 | -0.081 | -0.034 | 0.006 | 0.250 | -0.003 | 0.133 | 0.054 | 1.000 | 0.438 | 0.201 | 0.179 | 0.186 | 0.235 | 0.462 | 0.390 |
| High Cholesterol | -0.007 | -0.017 | -0.038 | -0.343 | 0.093 | 0.439 | 0.048 | 0.006 | -0.010 | -0.024 | -0.025 | 0.010 | 0.214 | -0.050 | 0.082 | 0.024 | 0.438 | 1.000 | 0.196 | 0.166 | 0.191 | 0.215 | 0.319 | 0.368 |
| Heart Failure | -0.007 | 0.036 | -0.022 | -0.125 | -0.001 | 0.192 | 0.076 | 0.000 | -0.032 | -0.055 | -0.011 | 0.009 | 0.086 | 0.001 | 0.029 | -0.004 | 0.201 | 0.196 | 1.000 | 0.239 | 0.389 | 0.279 | 0.282 | 0.174 |
| Stroke | -0.005 | 0.027 | -0.012 | -0.119 | 0.002 | 0.182 | 0.056 | 0.008 | -0.041 | -0.047 | -0.013 | 0.002 | 0.082 | 0.013 | 0.041 | 0.010 | 0.179 | 0.166 | 0.239 | 1.000 | 0.239 | 0.163 | 0.193 | 0.146 |
| Heart Attack | -0.045 | 0.008 | -0.027 | -0.121 | -0.012 | 0.198 | 0.045 | 0.006 | -0.023 | -0.047 | -0.014 | 0.011 | 0.089 | 0.002 | 0.053 | 0.013 | 0.186 | 0.191 | 0.389 | 0.239 | 1.000 | 0.231 | 0.296 | 0.149 |
| Other Heart Cond. | 0.012 | -0.010 | -0.026 | -0.136 | 0.021 | 0.221 | 0.052 | 0.006 | -0.011 | -0.050 | -0.019 | 0.014 | 0.129 | 0.016 | 0.072 | 0.049 | 0.235 | 0.215 | 0.279 | 0.163 | 0.231 | 1.000 | 0.342 | 0.194 |
| Beta Blockers | -0.014 | 0.035 | -0.050 | -0.246 | 0.021 | 0.362 | 0.054 | 0.028 | -0.015 | -0.062 | -0.025 | 0.013 | 0.163 | -0.028 | 0.060 | 0.008 | 0.462 | 0.319 | 0.282 | 0.193 | 0.296 | 0.342 | 1.000 | 0.286 |
| Diabetes | 0.025 | 0.056 | -0.012 | -0.238 | 0.077 | 0.302 | 0.068 | 0.031 | -0.022 | -0.092 | -0.027 | 0.021 | 0.167 | -0.006 | 0.059 | 0.026 | 0.390 | 0.368 | 0.174 | 0.146 | 0.149 | 0.194 | 0.286 | 1.000 |
| Bronchitis | 0.038 | -0.023 | 0.001 | 0.026 | -0.068 | 0.004 | 0.059 | -0.015 | -0.032 | -0.041 | 0.009 | 0.020 | -0.021 | 0.053 | -0.041 | 0.006 | 0.043 | 0.026 | 0.082 | 0.063 | 0.069 | 0.076 | 0.054 | 0.057 |
| Asthma | 0.026 | 0.043 | 0.029 | 0.061 | -0.037 | -0.056 | 0.038 | -0.010 | -0.037 | -0.028 | 0.006 | 0.009 | -0.026 | 0.036 | -0.017 | 0.023 | 0.029 | 0.005 | 0.041 | 0.033 | 0.019 | 0.061 | 0.012 | 0.046 |
| COPD | 0.026 | -0.011 | -0.036 | -0.195 | 0.009 | 0.288 | 0.103 | 0.016 | -0.047 | -0.088 | -0.015 | 0.007 | 0.164 | 0.074 | 0.073 | 0.041 | 0.228 | 0.215 | 0.262 | 0.192 | 0.209 | 0.209 | 0.220 | 0.174 |
| Emphysema | 0.001 | 0.002 | -0.020 | -0.113 | -0.010 | 0.187 | 0.076 | 0.001 | -0.031 | -0.062 | -0.008 | 0.006 | 0.109 | 0.058 | 0.053 | 0.033 | 0.130 | 0.142 | 0.216 | 0.133 | 0.156 | 0.155 | 0.138 | 0.119 |
| Other Respiratory Cond. | 0.043 | -0.019 | -0.012 | -0.116 | 0.067 | 0.141 | 0.030 | 0.003 | -0.004 | -0.020 | -0.017 | -0.003 | 0.109 | 0.027 | 0.071 | 0.051 | 0.144 | 0.134 | 0.144 | 0.090 | 0.110 | 0.189 | 0.142 | 0.132 |
| Cigs / 30d | 0.002 | 0.020 | -0.048 | -0.134 | 0.162 | 0.116 | 0.090 | 0.053 | -0.054 | -0.154 | -0.038 | 0.012 | 0.428 | 0.341 | 0.272 | 0.239 | 0.159 | 0.101 | 0.057 | 0.071 | 0.070 | 0.077 | 0.095 | 0.107 |
| Vape / 30d | -0.005 | -0.056 | 0.015 | 0.158 | -0.047 | -0.123 | 0.019 | 0.020 | -0.017 | -0.038 | -0.001 | 0.004 | 0.180 | 0.379 | 0.182 | 0.219 | -0.052 | -0.073 | -0.024 | -0.026 | -0.029 | -0.021 | -0.051 | -0.040 |
| Smoke Marijuana / 30d | -0.030 | 0.045 | 0.014 | 0.113 | 0.008 | -0.085 | 0.044 | 0.050 | -0.034 | -0.081 | -0.018 | 0.000 | 0.209 | 0.312 | 0.254 | 0.428 | -0.007 | -0.047 | -0.009 | -0.002 | -0.010 | 0.003 | -0.033 | -0.020 |
| Vape Marijuana / 30d | -0.007 | -0.065 | 0.018 | 0.107 | -0.017 | -0.095 | -0.005 | 0.019 | 0.000 | -0.004 | -0.003 | 0.003 | 0.106 | 0.211 | 0.138 | 0.257 | -0.035 | -0.051 | -0.024 | -0.013 | -0.022 | -0.008 | -0.037 | -0.028 |
| Other Marijuana / 30d | 0.021 | -0.042 | 0.008 | 0.025 | 0.034 | -0.027 | -0.015 | -0.006 | 0.007 | 0.030 | -0.003 | -0.005 | 0.086 | 0.092 | 0.110 | 0.210 | 0.002 | 0.002 | 0.001 | -0.011 | 0.007 | 0.016 | -0.012 | 0.003 |
| Cigs # Days | 0.019 | 0.021 | -0.054 | -0.181 | 0.185 | 0.147 | 0.095 | 0.053 | -0.055 | -0.155 | -0.037 | 0.013 | 0.383 | 0.297 | 0.235 | 0.203 | 0.179 | 0.126 | 0.067 | 0.085 | 0.082 | 0.087 | 0.110 | 0.122 |
| Vape # Days | -0.011 | -0.063 | 0.003 | 0.127 | -0.033 | -0.094 | 0.011 | 0.026 | -0.012 | -0.032 | -0.001 | 0.001 | 0.174 | 0.304 | 0.163 | 0.182 | -0.041 | -0.058 | -0.024 | -0.022 | -0.022 | -0.017 | -0.037 | -0.037 |
| Marijuana # Days | -0.035 | 0.020 | 0.012 | 0.087 | 0.028 | -0.070 | 0.047 | 0.048 | -0.025 | -0.082 | -0.020 | -0.001 | 0.221 | 0.290 | 0.249 | 0.379 | 0.009 | -0.035 | -0.007 | -0.003 | -0.004 | 0.016 | -0.026 | -0.004 |
| Wheezing / Life | 0.032 | -0.001 | -0.021 | -0.070 | 0.046 | 0.082 | 0.066 | 0.014 | -0.028 | -0.074 | -0.010 | 0.010 | 0.161 | 0.159 | 0.107 | 0.152 | 0.149 | 0.106 | 0.106 | 0.074 | 0.085 | 0.130 | 0.111 | 0.124 |
| Wheezing / 12mo | 0.044 | -0.010 | -0.012 | -0.094 | 0.051 | 0.102 | 0.069 | 0.022 | -0.024 | -0.072 | -0.018 | 0.006 | 0.153 | 0.126 | 0.089 | 0.109 | 0.140 | 0.101 | 0.130 | 0.080 | 0.089 | 0.128 | 0.112 | 0.108 |
| # Wheezing Attacks: 4-12 | 0.029 | -0.009 | -0.014 | -0.047 | 0.036 | 0.046 | 0.029 | 0.017 | -0.016 | -0.024 | -0.016 | -0.004 | 0.072 | 0.054 | 0.048 | 0.052 | 0.072 | 0.051 | 0.059 | 0.039 | 0.048 | 0.073 | 0.056 | 0.055 |
| # Wheezing Attacks: > 12 | 0.020 | -0.036 | -0.013 | -0.088 | 0.043 | 0.097 | 0.048 | 0.014 | -0.016 | -0.049 | -0.002 | 0.005 | 0.101 | 0.075 | 0.059 | 0.057 | 0.103 | 0.081 | 0.100 | 0.066 | 0.080 | 0.107 | 0.095 | 0.082 |
| Wheezing Disturbed Sleep: < 1 / Week | 0.032 | 0.014 | 0.006 | -0.035 | 0.029 | 0.034 | 0.039 | 0.005 | -0.012 | -0.037 | -0.014 | 0.005 | 0.064 | 0.055 | 0.039 | 0.042 | 0.068 | 0.042 | 0.044 | 0.036 | 0.031 | 0.065 | 0.042 | 0.060 |
| Wheezing Disturbed Sleep: ≥ 1 / Week | 0.023 | 0.012 | -0.005 | -0.066 | 0.038 | 0.075 | 0.051 | 0.013 | -0.029 | -0.061 | -0.018 | 0.011 | 0.093 | 0.068 | 0.061 | 0.050 | 0.112 | 0.073 | 0.131 | 0.083 | 0.083 | 0.100 | 0.083 | 0.086 |
| Wheezing Limited Speech | 0.039 | 0.003 | -0.002 | -0.041 | 0.017 | 0.047 | 0.051 | 0.010 | -0.035 | -0.045 | -0.005 | 0.010 | 0.060 | 0.056 | 0.037 | 0.042 | 0.067 | 0.044 | 0.090 | 0.080 | 0.072 | 0.098 | 0.060 | 0.054 |
| Wheezing From Exercise | 0.039 | -0.027 | -0.004 | -0.024 | 0.014 | 0.028 | 0.057 | 0.008 | -0.027 | -0.049 | -0.012 | 0.007 | 0.079 | 0.103 | 0.054 | 0.087 | 0.089 | 0.055 | 0.087 | 0.057 | 0.065 | 0.099 | 0.066 | 0.075 |
| Dry Cough at Night | 0.034 | 0.005 | 0.005 | -0.062 | 0.037 | 0.077 | 0.057 | 0.018 | -0.020 | -0.052 | -0.022 | 0.004 | 0.109 | 0.085 | 0.066 | 0.084 | 0.111 | 0.087 | 0.086 | 0.053 | 0.065 | 0.085 | 0.074 | 0.099 |
| Respiratory Index (0-9) | 0.054 | -0.011 | -0.013 | -0.103 | 0.060 | 0.117 | 0.090 | 0.023 | -0.039 | -0.092 | -0.022 | 0.011 | 0.181 | 0.161 | 0.114 | 0.142 | 0.182 | 0.129 | 0.162 | 0.108 | 0.120 | 0.171 | 0.139 | 0.149 |

|  | Bronchitis | Asthma | COPD | Emphysema | Other Respiratory Cond. | Cigs / 30d | Vape / 30d | Smoke Marijuana / 30d | Vape Marijuana / 30d | Other Marijuana / 30d | Cigs # Days | Vape # Days | Marijuana # Days | Wheezing / Life | Wheezing / 12mo | # Wheezing Attacks: 4-12 | # Wheezing Attacks: > 12 | Wheezing Disturbed Sleep: < 1 / Week | Wheezing Disturbed Sleep: ≥ 1 / Week | Wheezing Limited Speech | Wheezing From Exercise | Dry Cough at Night | Respiratory Index (0-9) |
| --- | --- | --- | --- | --- | --- | --- | --- | --- | --- | --- | --- | --- | --- | --- | --- | --- | --- | --- | --- | --- | --- | --- | --- |
| Female | 0.038 | 0.026 | 0.026 | 0.001 | 0.043 | 0.002 | -0.005 | -0.030 | -0.007 | 0.021 | 0.019 | -0.011 | -0.035 | 0.032 | 0.044 | 0.029 | 0.020 | 0.032 | 0.023 | 0.039 | 0.039 | 0.034 | 0.054 |
| Black | -0.023 | 0.043 | -0.011 | 0.002 | -0.019 | 0.020 | -0.056 | 0.045 | -0.065 | -0.042 | 0.021 | -0.063 | 0.020 | -0.001 | -0.010 | -0.009 | -0.036 | 0.014 | 0.012 | 0.003 | -0.027 | 0.005 | -0.011 |
| Other Race | 0.001 | 0.029 | -0.036 | -0.020 | -0.012 | -0.048 | 0.015 | 0.014 | 0.018 | 0.008 | -0.054 | 0.003 | 0.012 | -0.021 | -0.012 | -0.014 | -0.013 | 0.006 | -0.005 | -0.002 | -0.004 | 0.005 | -0.013 |
| 18-34 | 0.026 | 0.061 | -0.195 | -0.113 | -0.116 | -0.134 | 0.158 | 0.113 | 0.107 | 0.025 | -0.181 | 0.127 | 0.087 | -0.070 | -0.094 | -0.047 | -0.088 | -0.035 | -0.066 | -0.041 | -0.024 | -0.062 | -0.103 |
| 35-54 | -0.068 | -0.037 | 0.009 | -0.010 | 0.067 | 0.162 | -0.047 | 0.008 | -0.017 | 0.034 | 0.185 | -0.033 | 0.028 | 0.046 | 0.051 | 0.036 | 0.043 | 0.029 | 0.038 | 0.017 | 0.014 | 0.037 | 0.060 |
| 55+ | 0.004 | -0.056 | 0.288 | 0.187 | 0.141 | 0.116 | -0.123 | -0.085 | -0.095 | -0.027 | 0.147 | -0.094 | -0.070 | 0.082 | 0.102 | 0.046 | 0.097 | 0.034 | 0.075 | 0.047 | 0.028 | 0.077 | 0.117 |
| $10k - 25k | 0.059 | 0.038 | 0.103 | 0.076 | 0.030 | 0.090 | 0.019 | 0.044 | -0.005 | -0.015 | 0.095 | 0.011 | 0.047 | 0.066 | 0.069 | 0.029 | 0.048 | 0.039 | 0.051 | 0.051 | 0.057 | 0.057 | 0.090 |
| $25k - 50k | -0.015 | -0.010 | 0.016 | 0.001 | 0.003 | 0.053 | 0.020 | 0.050 | 0.019 | -0.006 | 0.053 | 0.026 | 0.048 | 0.014 | 0.022 | 0.017 | 0.014 | 0.005 | 0.013 | 0.010 | 0.008 | 0.018 | 0.023 |
| $50k - 100k | -0.032 | -0.037 | -0.047 | -0.031 | -0.004 | -0.054 | -0.017 | -0.034 | 0.000 | 0.007 | -0.055 | -0.012 | -0.025 | -0.028 | -0.024 | -0.016 | -0.016 | -0.012 | -0.029 | -0.035 | -0.027 | -0.020 | -0.039 |
| > $100k | -0.041 | -0.028 | -0.088 | -0.062 | -0.020 | -0.154 | -0.038 | -0.081 | -0.004 | 0.030 | -0.155 | -0.032 | -0.082 | -0.074 | -0.072 | -0.024 | -0.049 | -0.037 | -0.061 | -0.045 | -0.049 | -0.052 | -0.092 |
| Probe: > $50k | 0.009 | 0.006 | -0.015 | -0.008 | -0.017 | -0.038 | -0.001 | -0.018 | -0.003 | -0.003 | -0.037 | -0.001 | -0.020 | -0.010 | -0.018 | -0.016 | -0.002 | -0.014 | -0.018 | -0.005 | -0.012 | -0.022 | -0.022 |
| Probe: < $50k | 0.020 | 0.009 | 0.007 | 0.006 | -0.003 | 0.012 | 0.004 | 0.000 | 0.003 | -0.005 | 0.013 | 0.001 | -0.001 | 0.010 | 0.006 | -0.004 | 0.005 | 0.005 | 0.011 | 0.010 | 0.007 | 0.004 | 0.011 |
| Cigs / Life | -0.021 | -0.026 | 0.164 | 0.109 | 0.109 | 0.428 | 0.180 | 0.209 | 0.106 | 0.086 | 0.383 | 0.174 | 0.221 | 0.161 | 0.153 | 0.072 | 0.101 | 0.064 | 0.093 | 0.060 | 0.079 | 0.109 | 0.181 |
| Vape / Life | 0.053 | 0.036 | 0.074 | 0.058 | 0.027 | 0.341 | 0.379 | 0.312 | 0.211 | 0.092 | 0.297 | 0.304 | 0.290 | 0.159 | 0.126 | 0.054 | 0.075 | 0.055 | 0.068 | 0.056 | 0.103 | 0.085 | 0.161 |
| Other Tobacco / Life | -0.041 | -0.017 | 0.073 | 0.053 | 0.071 | 0.272 | 0.182 | 0.254 | 0.138 | 0.110 | 0.235 | 0.163 | 0.249 | 0.107 | 0.089 | 0.048 | 0.059 | 0.039 | 0.061 | 0.037 | 0.054 | 0.066 | 0.114 |
| Marijuana / Life | 0.006 | 0.023 | 0.041 | 0.033 | 0.051 | 0.239 | 0.219 | 0.428 | 0.257 | 0.210 | 0.203 | 0.182 | 0.379 | 0.152 | 0.109 | 0.052 | 0.057 | 0.042 | 0.050 | 0.042 | 0.087 | 0.084 | 0.142 |
| High Blood Pressure | 0.043 | 0.029 | 0.228 | 0.130 | 0.144 | 0.159 | -0.052 | -0.007 | -0.035 | 0.002 | 0.179 | -0.041 | 0.009 | 0.149 | 0.140 | 0.072 | 0.103 | 0.068 | 0.112 | 0.067 | 0.089 | 0.111 | 0.182 |
| High Cholesterol | 0.026 | 0.005 | 0.215 | 0.142 | 0.134 | 0.101 | -0.073 | -0.047 | -0.051 | 0.002 | 0.126 | -0.058 | -0.035 | 0.106 | 0.101 | 0.051 | 0.081 | 0.042 | 0.073 | 0.044 | 0.055 | 0.087 | 0.129 |
| Heart Failure | 0.082 | 0.041 | 0.262 | 0.216 | 0.144 | 0.057 | -0.024 | -0.009 | -0.024 | 0.001 | 0.067 | -0.024 | -0.007 | 0.106 | 0.130 | 0.059 | 0.100 | 0.044 | 0.131 | 0.090 | 0.087 | 0.086 | 0.162 |
| Stroke | 0.063 | 0.033 | 0.192 | 0.133 | 0.090 | 0.071 | -0.026 | -0.002 | -0.013 | -0.011 | 0.085 | -0.022 | -0.003 | 0.074 | 0.080 | 0.039 | 0.066 | 0.036 | 0.083 | 0.080 | 0.057 | 0.053 | 0.108 |
| Heart Attack | 0.069 | 0.019 | 0.209 | 0.156 | 0.110 | 0.070 | -0.029 | -0.010 | -0.022 | 0.007 | 0.082 | -0.022 | -0.004 | 0.085 | 0.089 | 0.048 | 0.080 | 0.031 | 0.083 | 0.072 | 0.065 | 0.065 | 0.120 |
| Other Heart Cond. | 0.076 | 0.061 | 0.209 | 0.155 | 0.189 | 0.077 | -0.021 | 0.003 | -0.008 | 0.016 | 0.087 | -0.017 | 0.016 | 0.130 | 0.128 | 0.073 | 0.107 | 0.065 | 0.100 | 0.098 | 0.099 | 0.085 | 0.171 |
| Beta Blockers | 0.054 | 0.012 | 0.220 | 0.138 | 0.142 | 0.095 | -0.051 | -0.033 | -0.037 | -0.012 | 0.110 | -0.037 | -0.026 | 0.111 | 0.112 | 0.056 | 0.095 | 0.042 | 0.083 | 0.060 | 0.066 | 0.074 | 0.139 |
| Diabetes | 0.057 | 0.046 | 0.174 | 0.119 | 0.132 | 0.107 | -0.040 | -0.020 | -0.028 | 0.003 | 0.122 | -0.037 | -0.004 | 0.124 | 0.108 | 0.055 | 0.082 | 0.060 | 0.086 | 0.054 | 0.075 | 0.099 | 0.149 |
| Bronchitis | 1.000 | 0.238 | 0.206 | 0.162 | 0.111 | 0.045 | 0.045 | 0.028 | 0.031 | 0.011 | 0.045 | 0.037 | 0.029 | 0.216 | 0.157 | 0.069 | 0.105 | 0.087 | 0.108 | 0.110 | 0.147 | 0.094 | 0.219 |
| Asthma | 0.238 | 1.000 | 0.100 | 0.085 | 0.081 | 0.000 | 0.028 | 0.032 | 0.024 | 0.039 | -0.001 | 0.018 | 0.033 | 0.382 | 0.254 | 0.139 | 0.125 | 0.151 | 0.130 | 0.154 | 0.237 | 0.095 | 0.337 |
| COPD | 0.206 | 0.100 | 1.000 | 0.472 | 0.198 | 0.204 | -0.018 | 0.007 | -0.025 | -0.007 | 0.230 | -0.011 | 0.014 | 0.206 | 0.262 | 0.123 | 0.244 | 0.093 | 0.220 | 0.166 | 0.189 | 0.141 | 0.318 |
| Emphysema | 0.162 | 0.085 | 0.472 | 1.000 | 0.152 | 0.143 | -0.003 | 0.018 | -0.017 | 0.004 | 0.159 | -0.004 | 0.023 | 0.125 | 0.162 | 0.060 | 0.185 | 0.056 | 0.183 | 0.123 | 0.134 | 0.087 | 0.215 |
| Other Respiratory Cond. | 0.111 | 0.081 | 0.198 | 0.152 | 1.000 | 0.077 | -0.015 | 0.007 | 0.001 | 0.031 | 0.087 | -0.018 | 0.020 | 0.192 | 0.183 | 0.087 | 0.149 | 0.089 | 0.150 | 0.134 | 0.127 | 0.128 | 0.242 |
| Cigs / 30d | 0.045 | 0.000 | 0.204 | 0.143 | 0.077 | 1.000 | 0.153 | 0.219 | 0.072 | 0.024 | 0.896 | 0.098 | 0.211 | 0.220 | 0.231 | 0.107 | 0.133 | 0.106 | 0.126 | 0.071 | 0.136 | 0.160 | 0.261 |
| Vape / 30d | 0.045 | 0.028 | -0.018 | -0.003 | -0.015 | 0.153 | 1.000 | 0.231 | 0.224 | 0.056 | 0.062 | 0.801 | 0.208 | 0.074 | 0.029 | 0.014 | 0.000 | 0.016 | -0.002 | 0.010 | 0.044 | 0.036 | 0.050 |
| Smoke Marijuana / 30d | 0.028 | 0.032 | 0.007 | 0.018 | 0.007 | 0.219 | 0.231 | 1.000 | 0.402 | 0.168 | 0.183 | 0.171 | 0.751 | 0.118 | 0.100 | 0.047 | 0.035 | 0.046 | 0.041 | 0.039 | 0.085 | 0.075 | 0.120 |
| Vape Marijuana / 30d | 0.031 | 0.024 | -0.025 | -0.017 | 0.001 | 0.072 | 0.224 | 0.402 | 1.000 | 0.198 | 0.037 | 0.187 | 0.474 | 0.069 | 0.058 | 0.023 | 0.012 | 0.028 | 0.007 | 0.024 | 0.064 | 0.047 | 0.069 |
| Other Marijuana / 30d | 0.011 | 0.039 | -0.007 | 0.004 | 0.031 | 0.024 | 0.056 | 0.168 | 0.198 | 1.000 | 0.012 | 0.041 | 0.286 | 0.059 | 0.072 | 0.038 | 0.043 | 0.038 | 0.031 | 0.036 | 0.062 | 0.034 | 0.080 |
| Cigs # Days | 0.045 | -0.001 | 0.230 | 0.159 | 0.087 | 0.896 | 0.062 | 0.183 | 0.037 | 0.012 | 1.000 | 0.014 | 0.187 | 0.233 | 0.255 | 0.124 | 0.152 | 0.112 | 0.145 | 0.083 | 0.149 | 0.175 | 0.287 |
| Vape # Days | 0.037 | 0.018 | -0.011 | -0.004 | -0.018 | 0.098 | 0.801 | 0.171 | 0.187 | 0.041 | 0.014 | 1.000 | 0.177 | 0.066 | 0.021 | 0.011 | -0.001 | 0.014 | -0.012 | -0.003 | 0.035 | 0.032 | 0.039 |
| Marijuana # Days | 0.029 | 0.033 | 0.014 | 0.023 | 0.020 | 0.211 | 0.208 | 0.751 | 0.474 | 0.286 | 0.187 | 0.177 | 1.000 | 0.134 | 0.126 | 0.049 | 0.061 | 0.053 | 0.054 | 0.048 | 0.102 | 0.084 | 0.146 |
| Wheezing / Life | 0.216 | 0.382 | 0.206 | 0.125 | 0.192 | 0.220 | 0.074 | 0.118 | 0.069 | 0.059 | 0.233 | 0.066 | 0.134 | 1.000 | 0.449 | 0.200 | 0.189 | 0.201 | 0.174 | 0.177 | 0.325 | 0.220 | 0.653 |
| Wheezing / 12mo | 0.157 | 0.254 | 0.262 | 0.162 | 0.183 | 0.231 | 0.029 | 0.100 | 0.058 | 0.072 | 0.255 | 0.021 | 0.126 | 0.449 | 1.000 | 0.446 | 0.420 | 0.447 | 0.388 | 0.394 | 0.565 | 0.316 | 0.825 |
| # Wheezing Attacks: 4-12 | 0.069 | 0.139 | 0.123 | 0.060 | 0.087 | 0.107 | 0.014 | 0.047 | 0.023 | 0.038 | 0.124 | 0.011 | 0.049 | 0.200 | 0.446 | 1.000 | -0.028 | 0.267 | 0.201 | 0.210 | 0.284 | 0.167 | 0.438 |
| # Wheezing Attacks: > 12 | 0.105 | 0.125 | 0.244 | 0.185 | 0.149 | 0.133 | 0.000 | 0.035 | 0.012 | 0.043 | 0.152 | -0.001 | 0.061 | 0.189 | 0.420 | -0.028 | 1.000 | 0.156 | 0.404 | 0.328 | 0.315 | 0.171 | 0.569 |
| Wheezing Disturbed Sleep: < 1 / Week | 0.087 | 0.151 | 0.093 | 0.056 | 0.089 | 0.106 | 0.016 | 0.046 | 0.028 | 0.038 | 0.112 | 0.014 | 0.053 | 0.201 | 0.447 | 0.267 | 0.156 | 1.000 | -0.026 | 0.231 | 0.270 | 0.180 | 0.435 |
| Wheezing Disturbed Sleep: ≥ 1 / Week | 0.108 | 0.130 | 0.220 | 0.183 | 0.150 | 0.126 | -0.002 | 0.041 | 0.007 | 0.031 | 0.145 | -0.012 | 0.054 | 0.174 | 0.388 | 0.201 | 0.404 | -0.026 | 1.000 | 0.341 | 0.286 | 0.206 | 0.557 |
| Wheezing Limited Speech | 0.110 | 0.154 | 0.166 | 0.123 | 0.134 | 0.071 | 0.010 | 0.039 | 0.024 | 0.036 | 0.083 | -0.003 | 0.048 | 0.177 | 0.394 | 0.210 | 0.328 | 0.231 | 0.341 | 1.000 | 0.328 | 0.189 | 0.517 |
| Wheezing From Exercise | 0.147 | 0.237 | 0.189 | 0.134 | 0.127 | 0.136 | 0.044 | 0.085 | 0.064 | 0.062 | 0.149 | 0.035 | 0.102 | 0.325 | 0.565 | 0.284 | 0.315 | 0.270 | 0.286 | 0.328 | 1.000 | 0.279 | 0.687 |
| Dry Cough at Night | 0.094 | 0.095 | 0.141 | 0.087 | 0.128 | 0.160 | 0.036 | 0.075 | 0.047 | 0.034 | 0.175 | 0.032 | 0.084 | 0.220 | 0.316 | 0.167 | 0.171 | 0.180 | 0.206 | 0.189 | 0.279 | 1.000 | 0.550 |
| Respiratory Index (0-9) | 0.219 | 0.337 | 0.318 | 0.215 | 0.242 | 0.261 | 0.050 | 0.120 | 0.069 | 0.080 | 0.287 | 0.039 | 0.146 | 0.653 | 0.825 | 0.438 | 0.569 | 0.435 | 0.557 | 0.517 | 0.687 | 0.550 | 1.000 |

Notes: Unweighted Pearson correlation coefficients are given. Only participants with a wave 4 cross-sectional weight are included. Listwise deletion was used for handling incomplete cases.
